# Supplementary material for: Therapeutic effects of PDGF-AB/BB against cellular senescence in human intervertebral disc
Source: eLife. 2025 Jul 16;13:RP103073. doi: 10.7554/eLife.103073 (PMC12266719; doi:10.7554/eLife.103073)

NP samples #1 #2 #3

Chemi

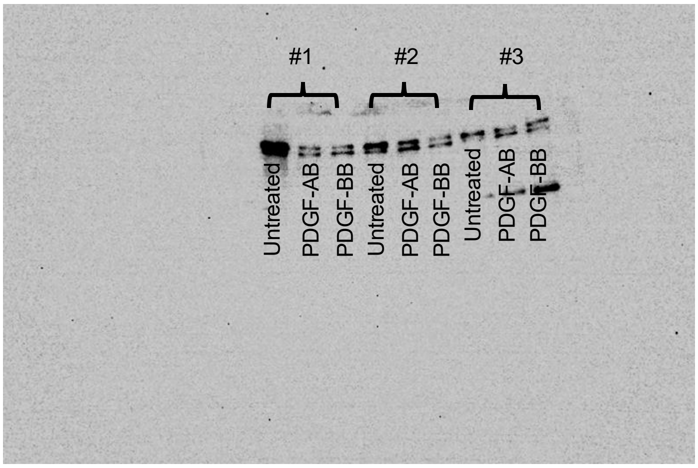

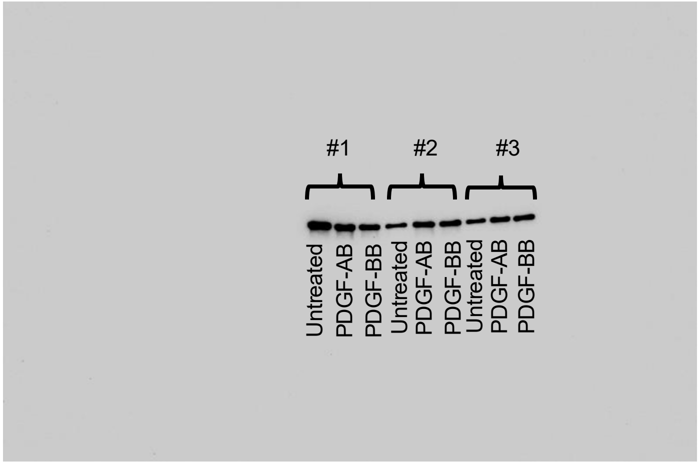

Overlay

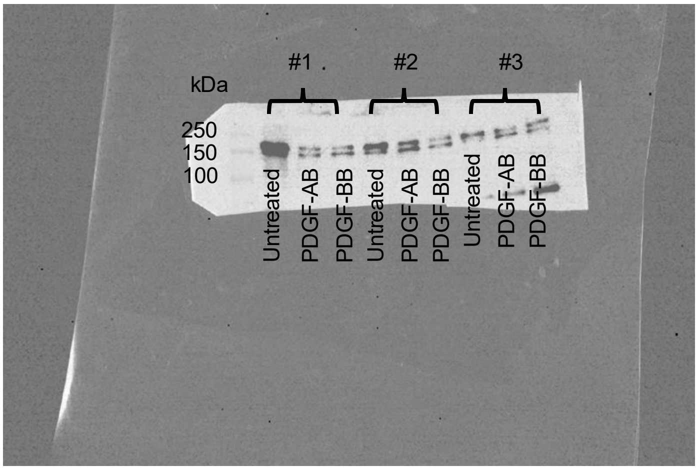

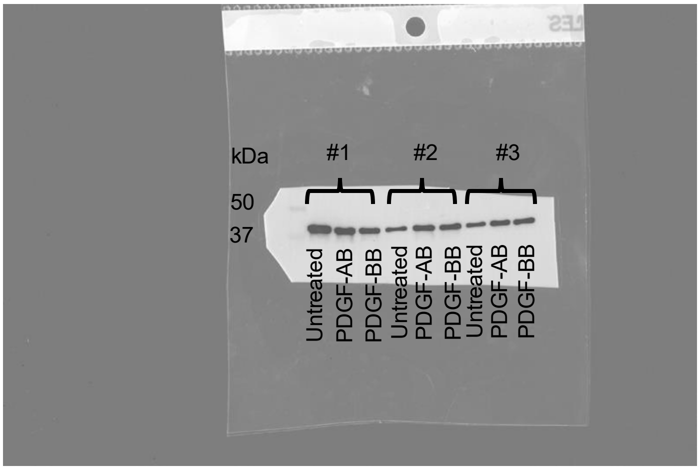

NP samples #4 #5

Chemi

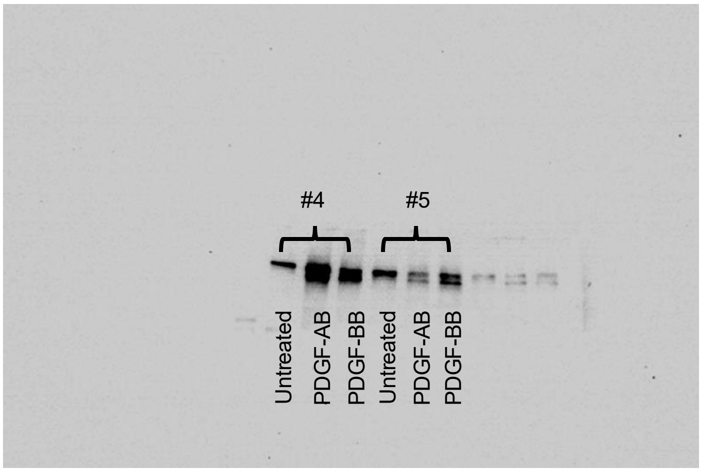

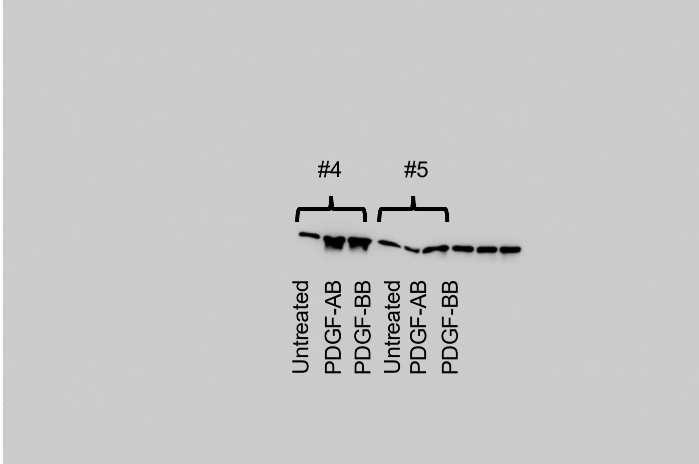

Overlay

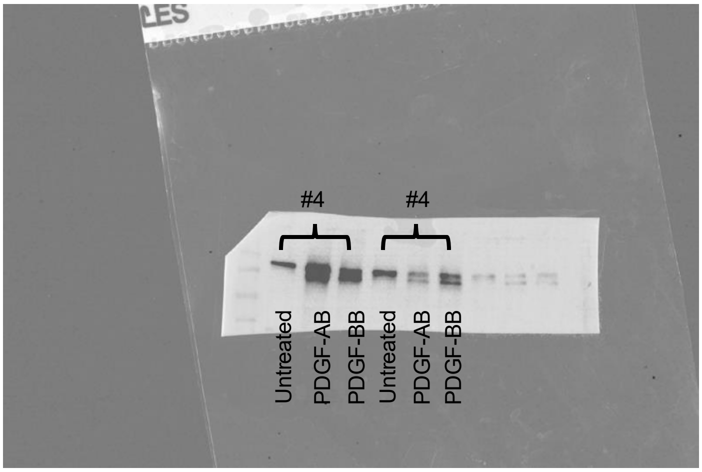

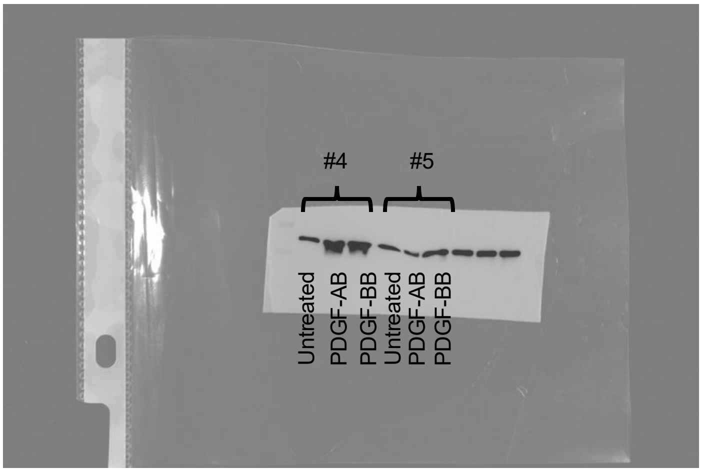

AF samples #1 #2 #3

Chemi

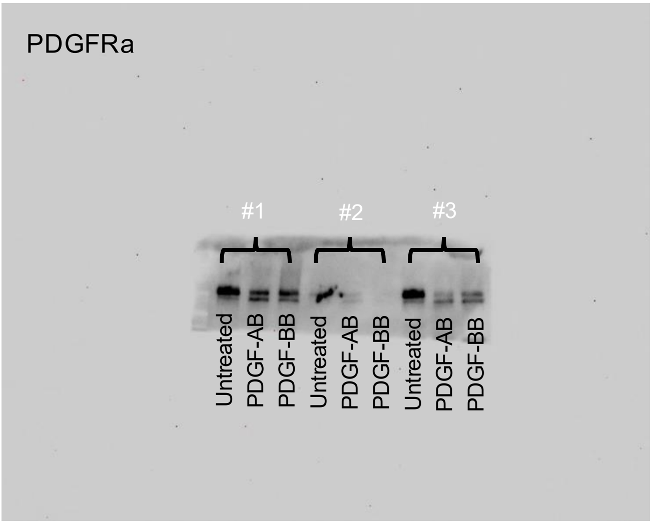

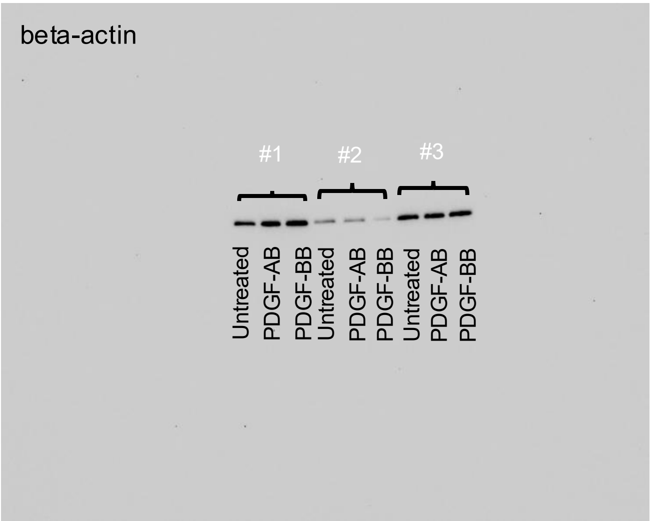

Overlay

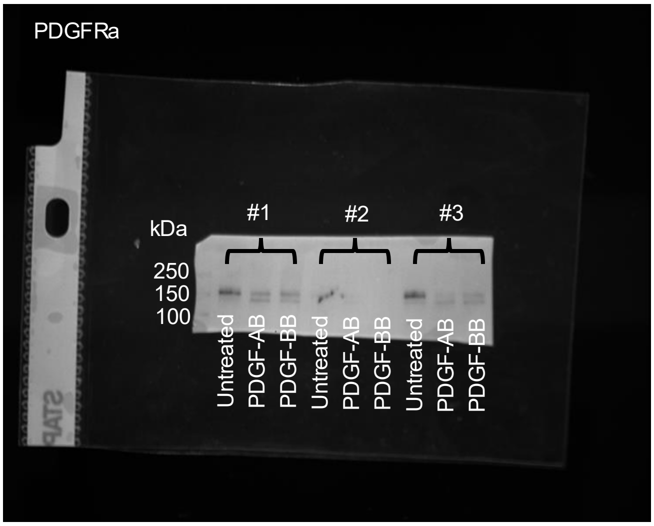

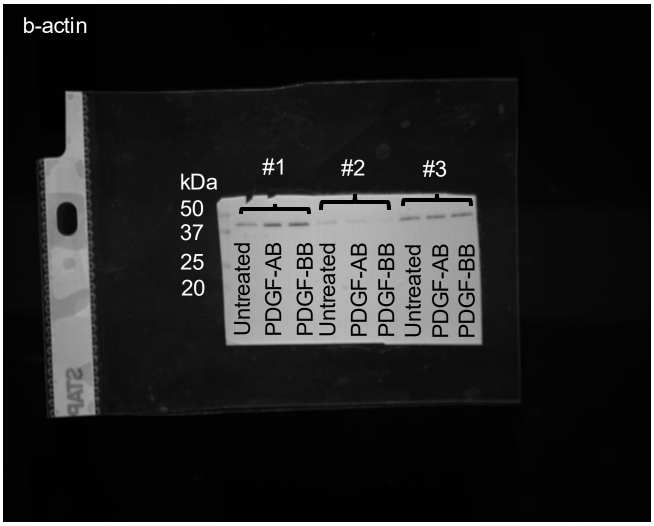

AF samples #4 #5 #6

Chemi

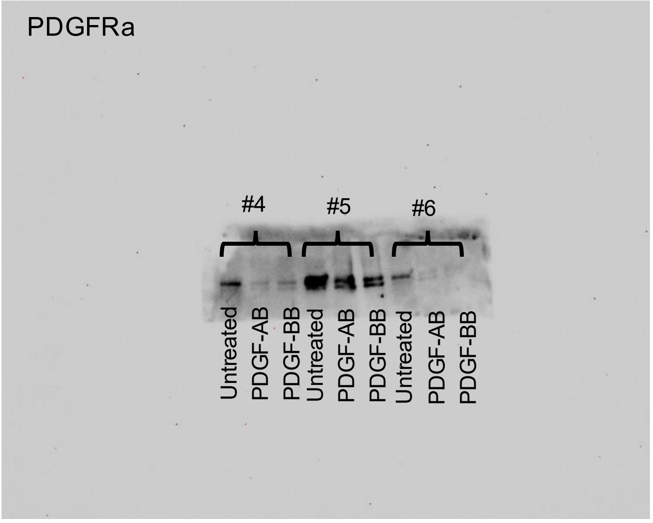

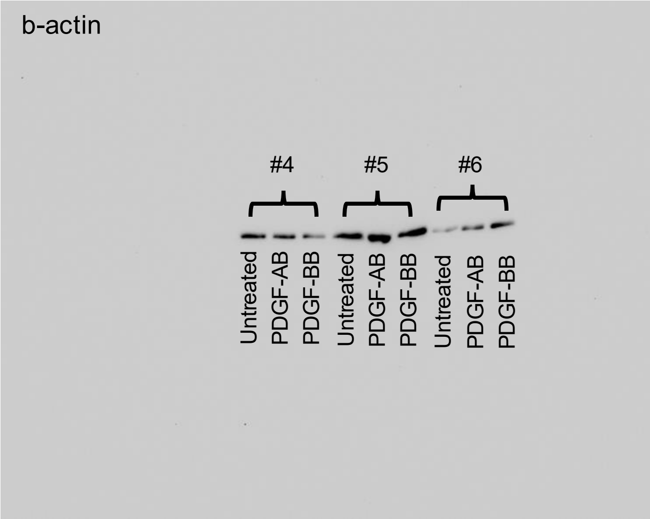

Overlay

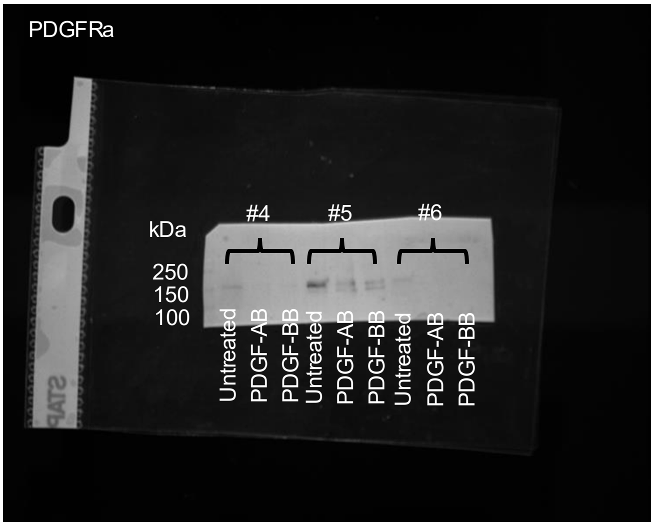

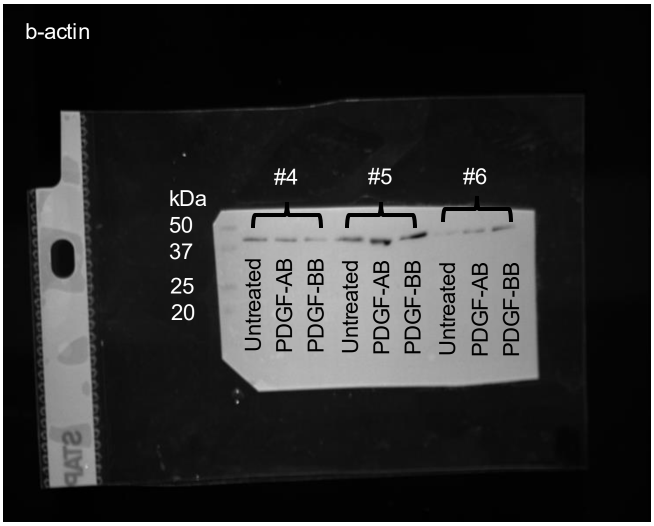

Supplement: Figure 3—figure supplement 1—source data 2. [file elife-103073-fig3-figsupp1-data2.zip › PDGFRA NP AF WB.pdf]
