# Supplementary figures and images for: Therapeutic effects of PDGF-AB/BB against cellular senescence in human intervertebral disc
Source: eLife. 2025 Jul 16;13:RP103073. doi: 10.7554/eLife.103073 (PMC12266719; doi:10.7554/eLife.103073)

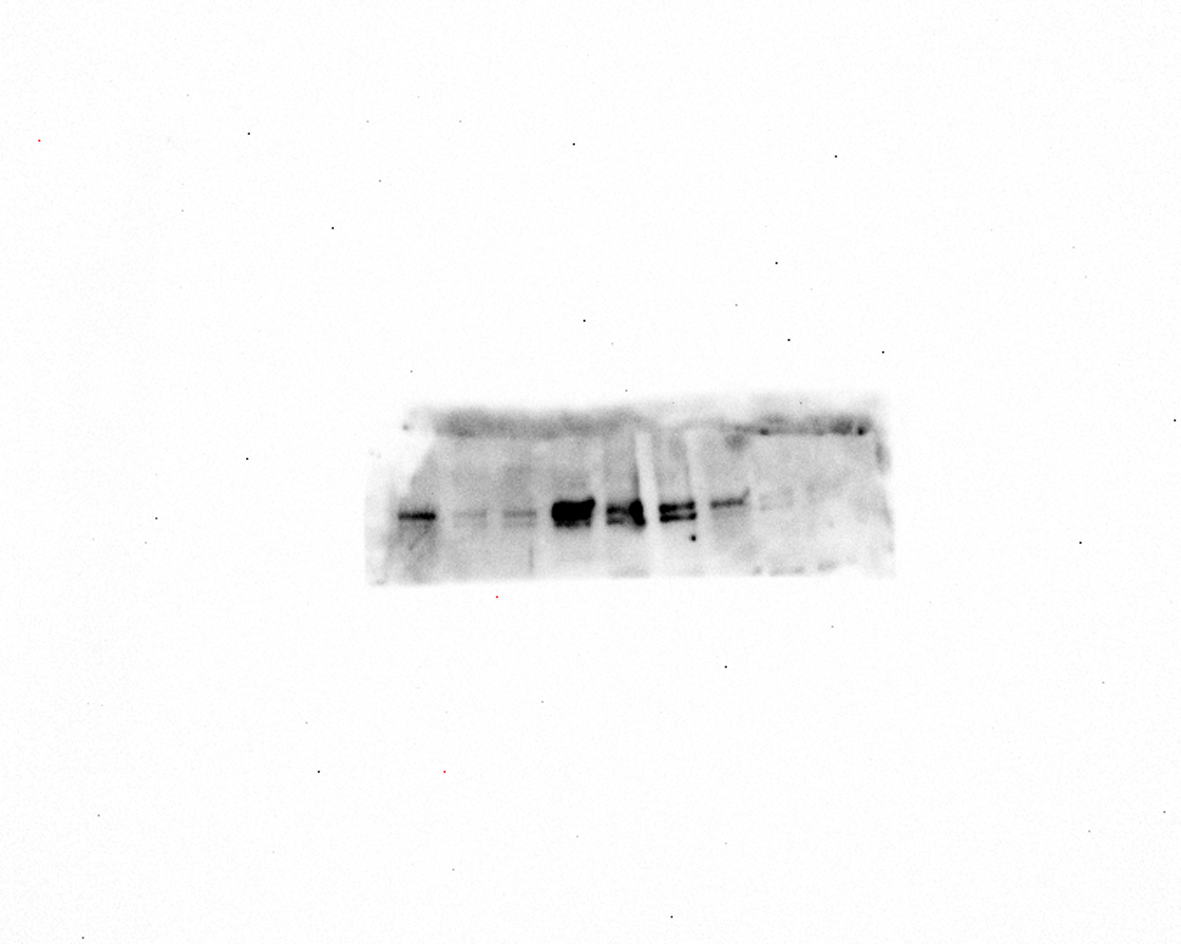

Supplement: Figure 3—figure supplement 1—source data 3. [file elife-103073-fig3-figsupp1-data3.zip › PDGFRA/PDGFRA AF#4 5 6(Chemi).tif]

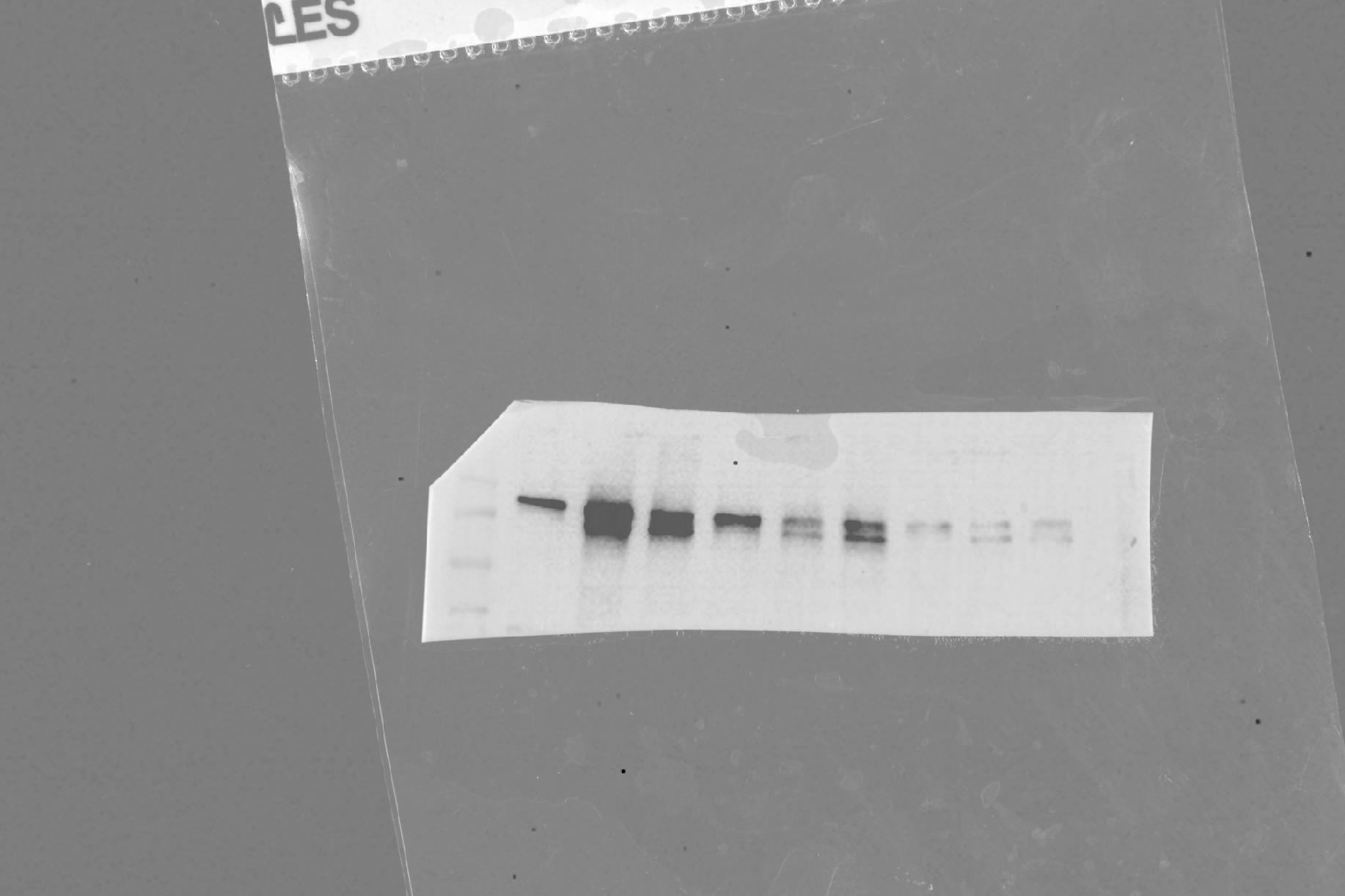

Supplement: Figure 3—figure supplement 1—source data 3. [file elife-103073-fig3-figsupp1-data3.zip › PDGFRA/PDGFRA NP#4 5(Composite).tif]

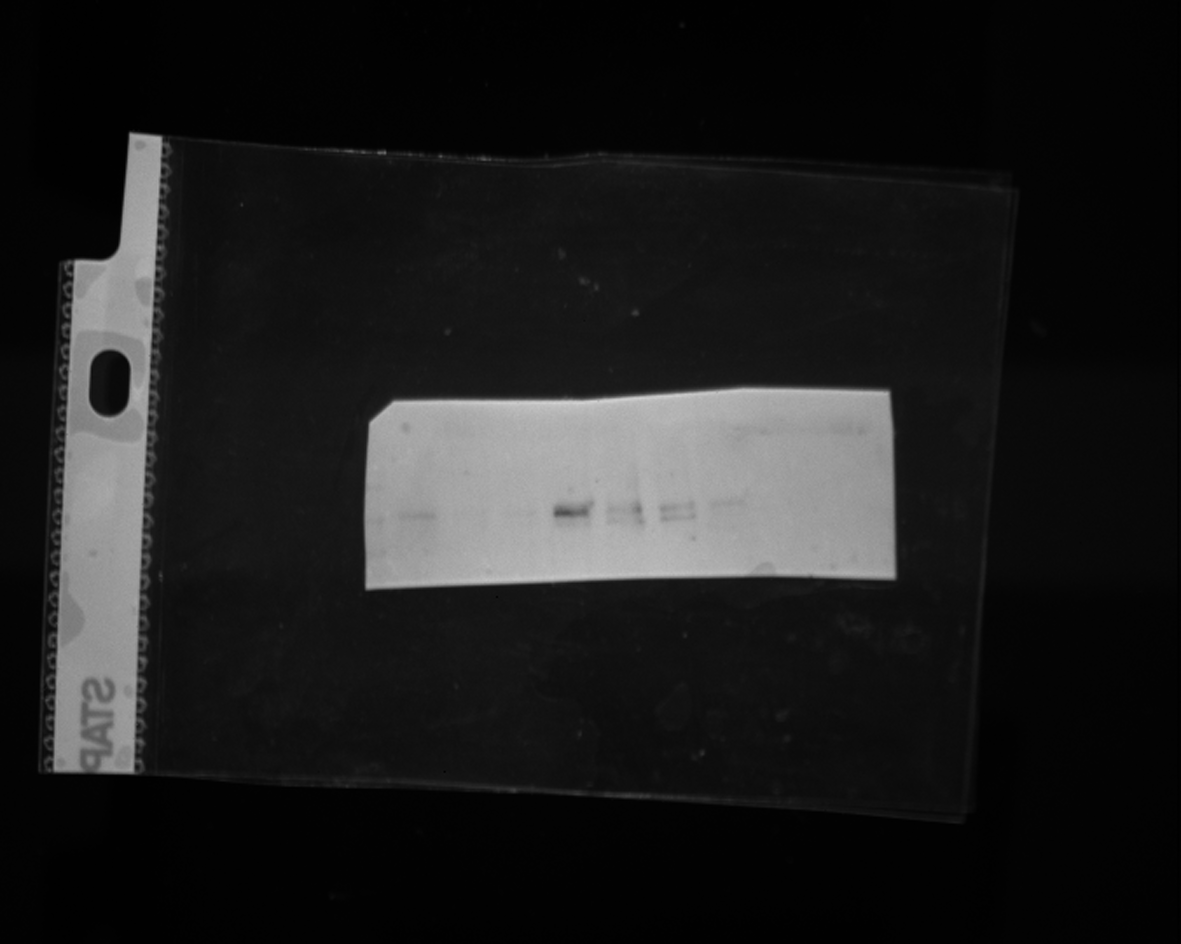

Supplement: Figure 3—figure supplement 1—source data 3. [file elife-103073-fig3-figsupp1-data3.zip › PDGFRA/PDGFRA AF#4 5 6(Overlay).tif]

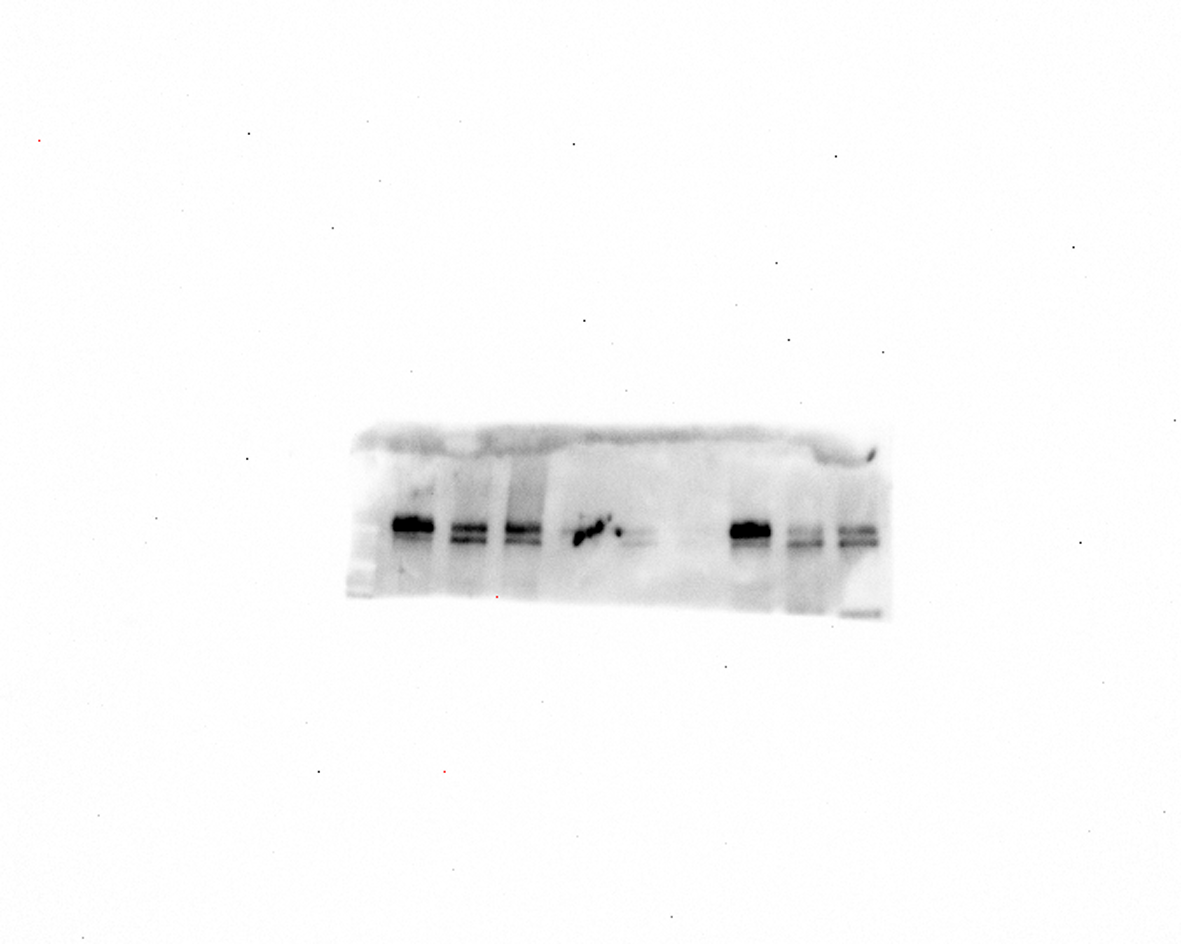

Supplement: Figure 3—figure supplement 1—source data 3. [file elife-103073-fig3-figsupp1-data3.zip › PDGFRA/PDGFRA AF#1 2 3(Chemi).tif]

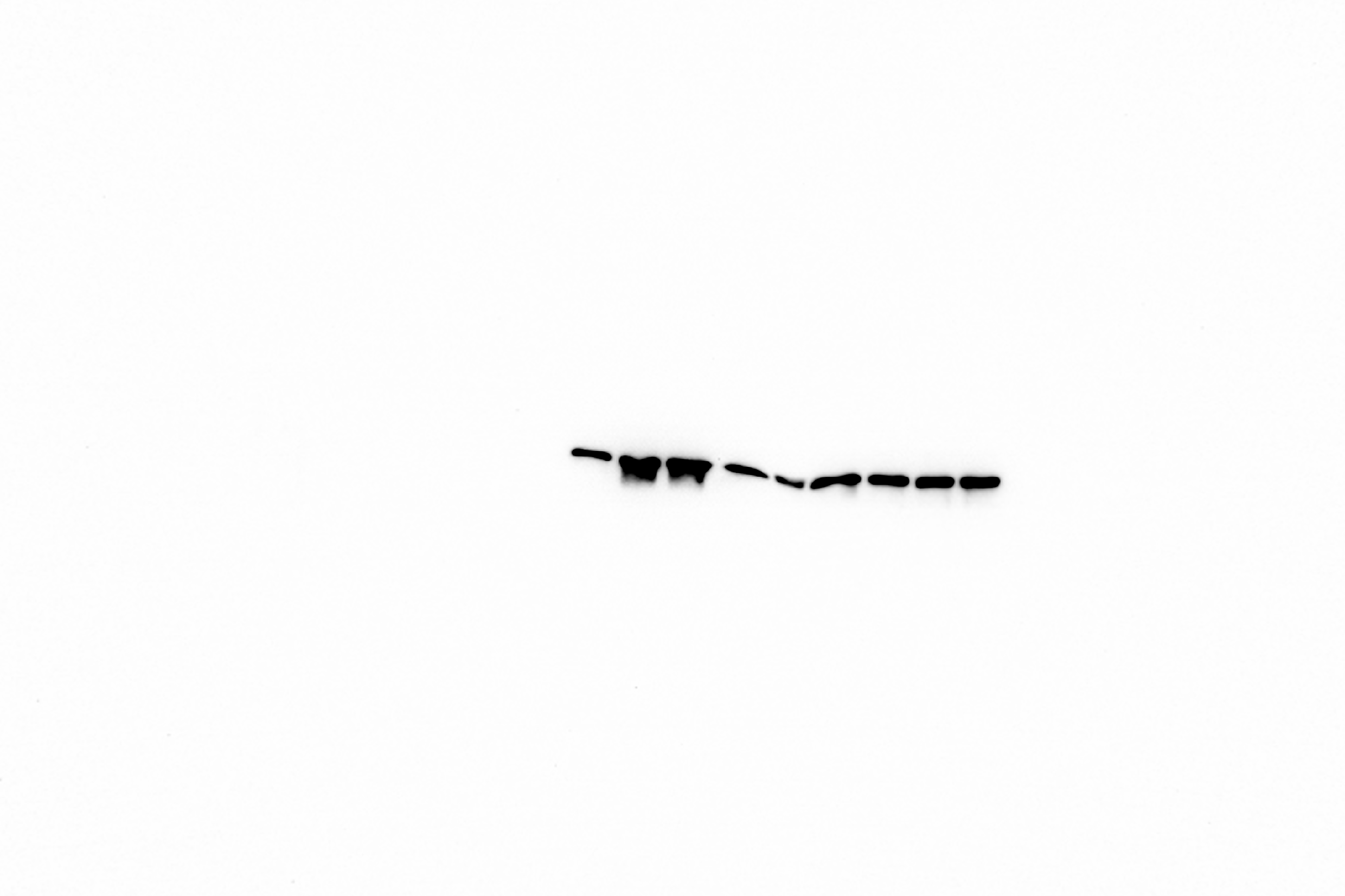

Supplement: Figure 3—figure supplement 1—source data 3. [file elife-103073-fig3-figsupp1-data3.zip › PDGFRA/b-actin NP#4 5(Chemiluminescence).tif]

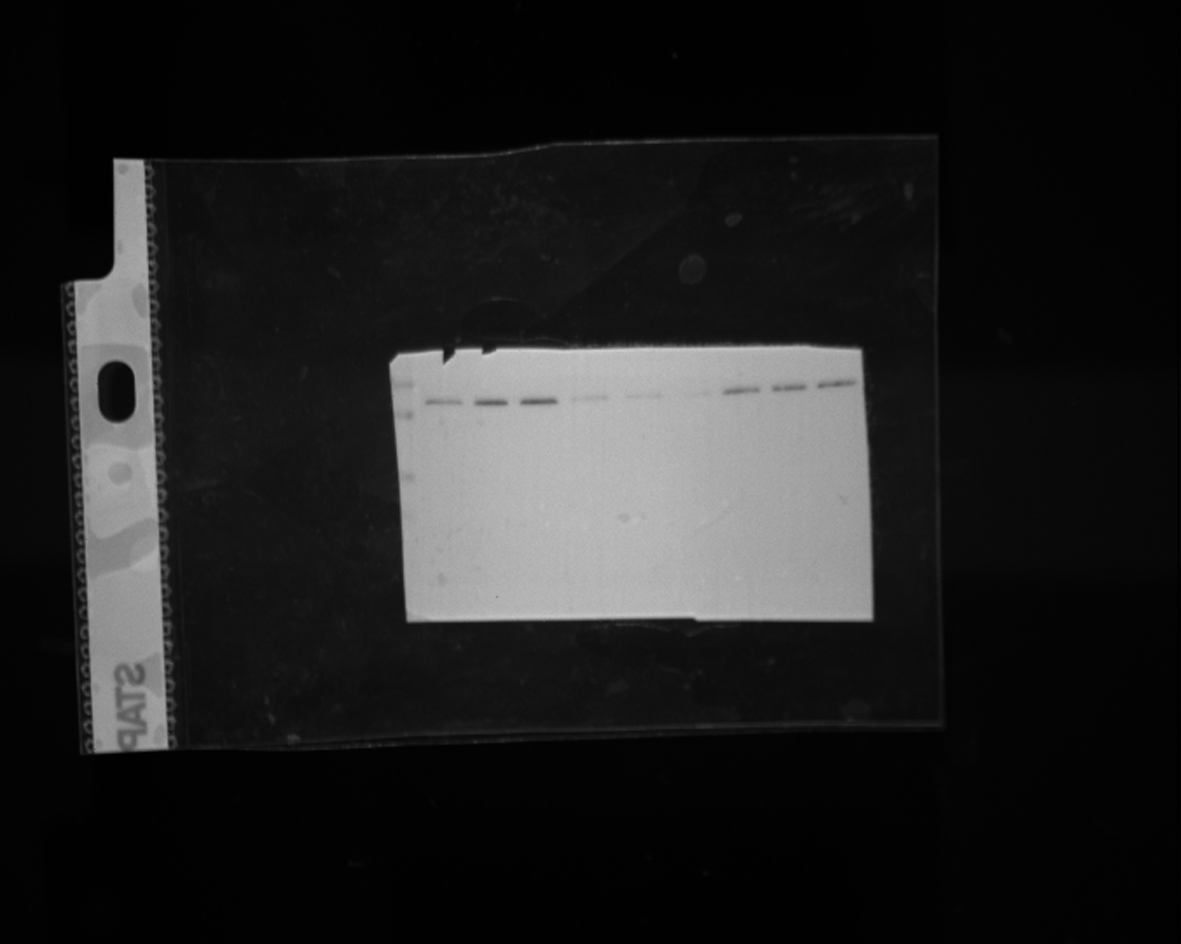

Supplement: Figure 3—figure supplement 1—source data 3. [file elife-103073-fig3-figsupp1-data3.zip › PDGFRA/b-actin AF#1 2 3(Overlay).tif]

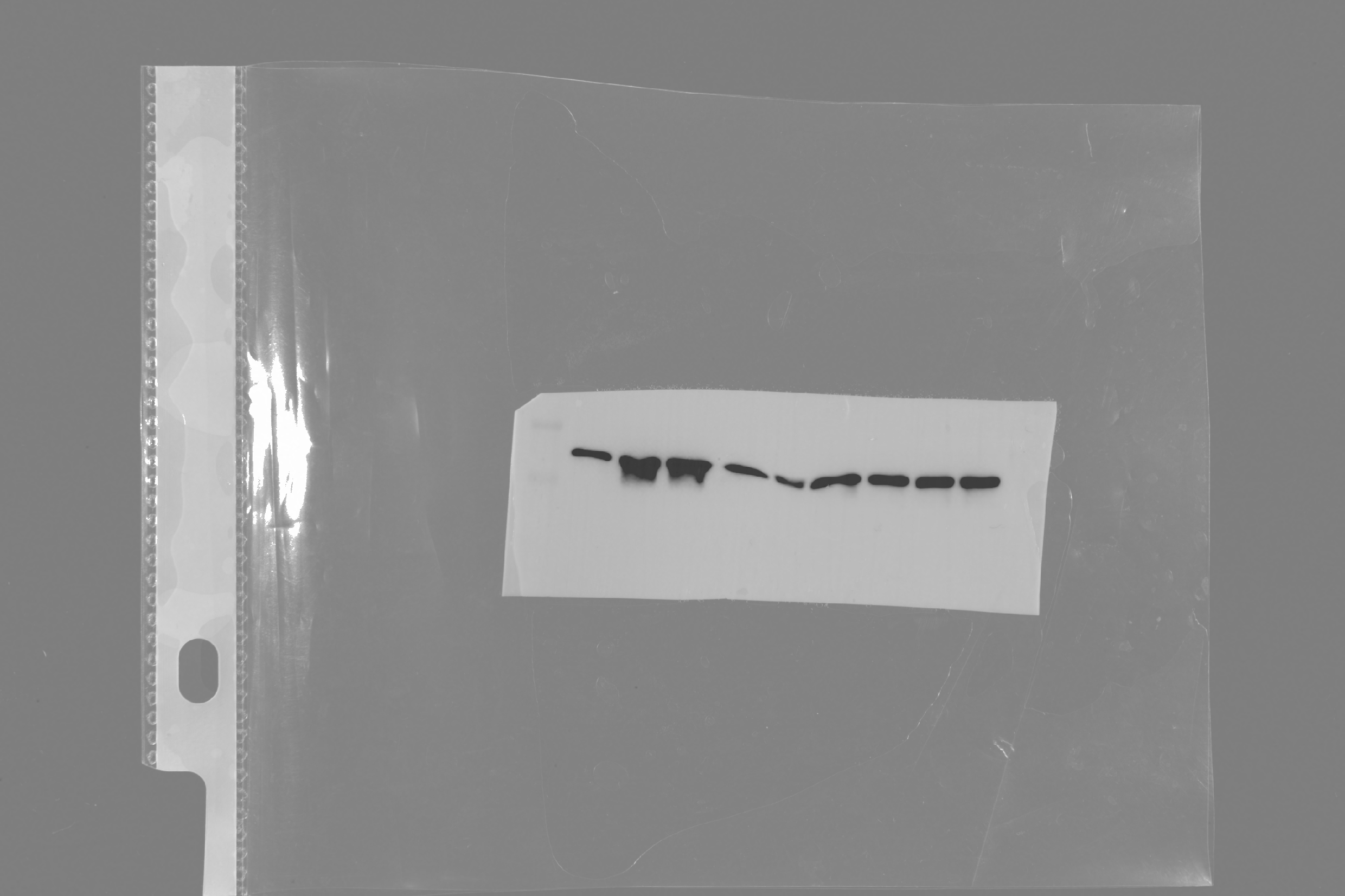

Supplement: Figure 3—figure supplement 1—source data 3. [file elife-103073-fig3-figsupp1-data3.zip › PDGFRA/b-actin NP#4 5(Composite).tif]

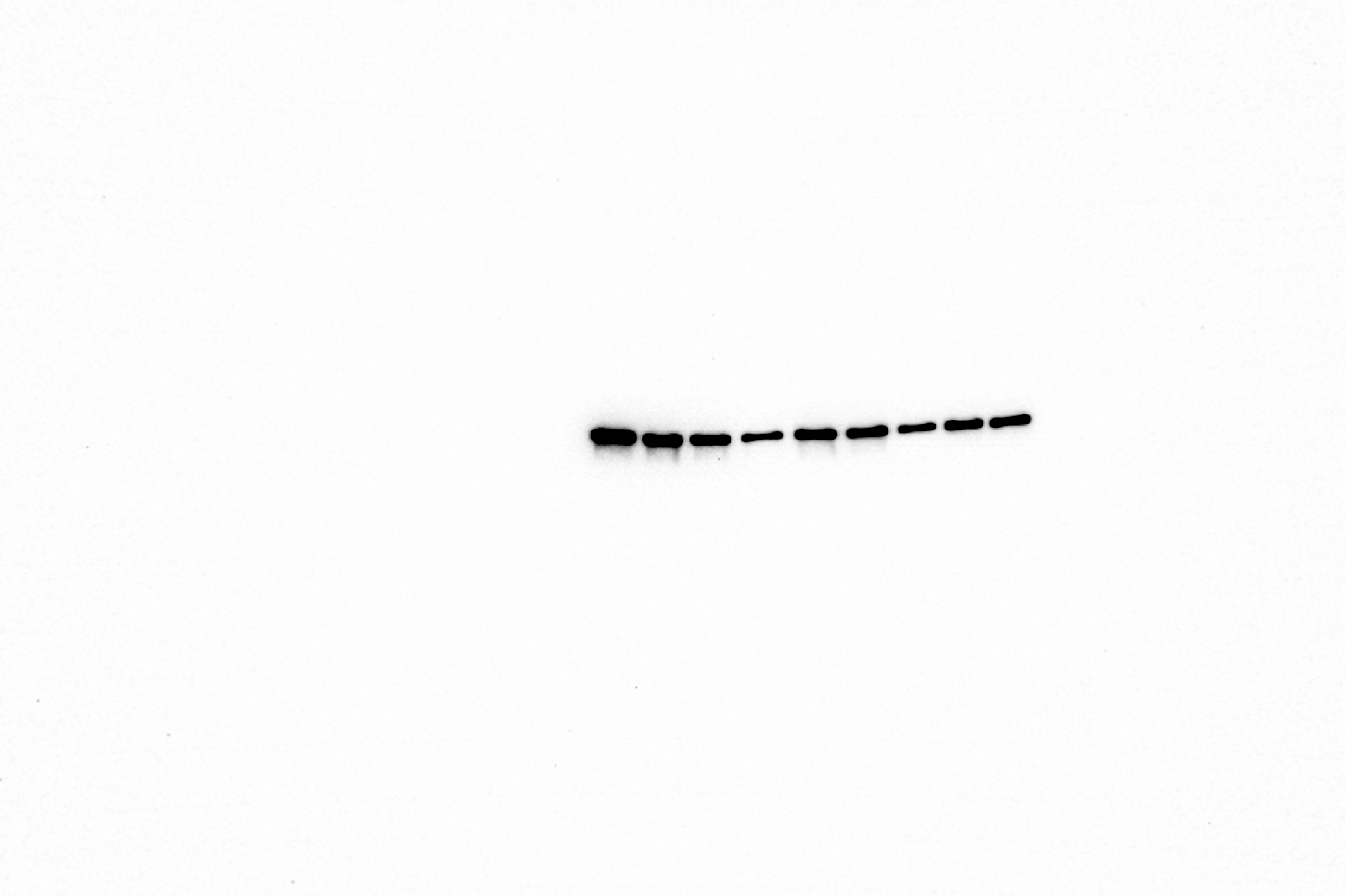

Supplement: Figure 3—figure supplement 1—source data 3. [file elife-103073-fig3-figsupp1-data3.zip › PDGFRA/b-actin NP#1 2 3(Chemiluminescence).tif]

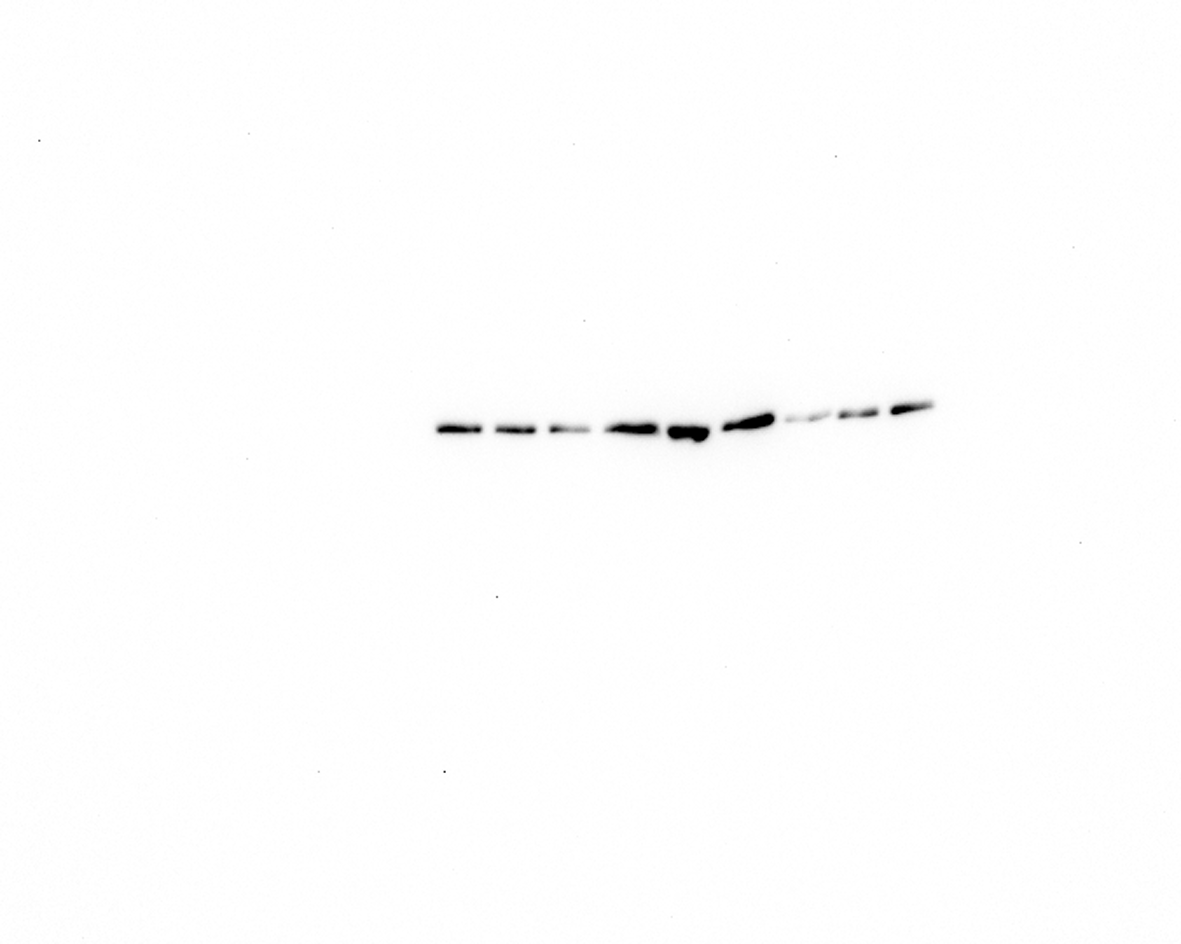

Supplement: Figure 3—figure supplement 1—source data 3. [file elife-103073-fig3-figsupp1-data3.zip › PDGFRA/b-actin AF#4 5 6(Chemi).tif]

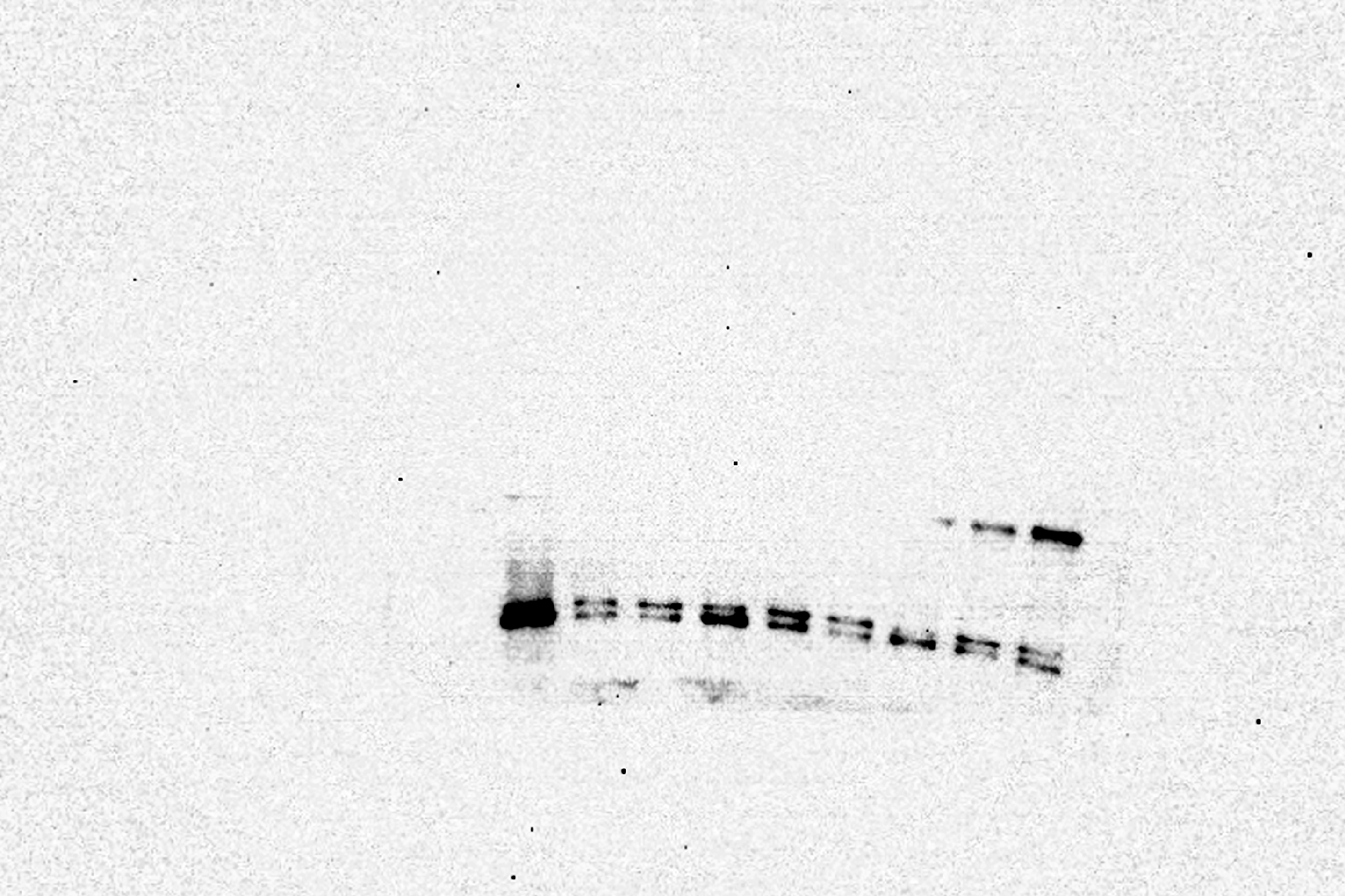

Supplement: Figure 3—figure supplement 1—source data 3. [file elife-103073-fig3-figsupp1-data3.zip › PDGFRA/PDGFRA NP#1 2 3(Chemiluminescence).tif]

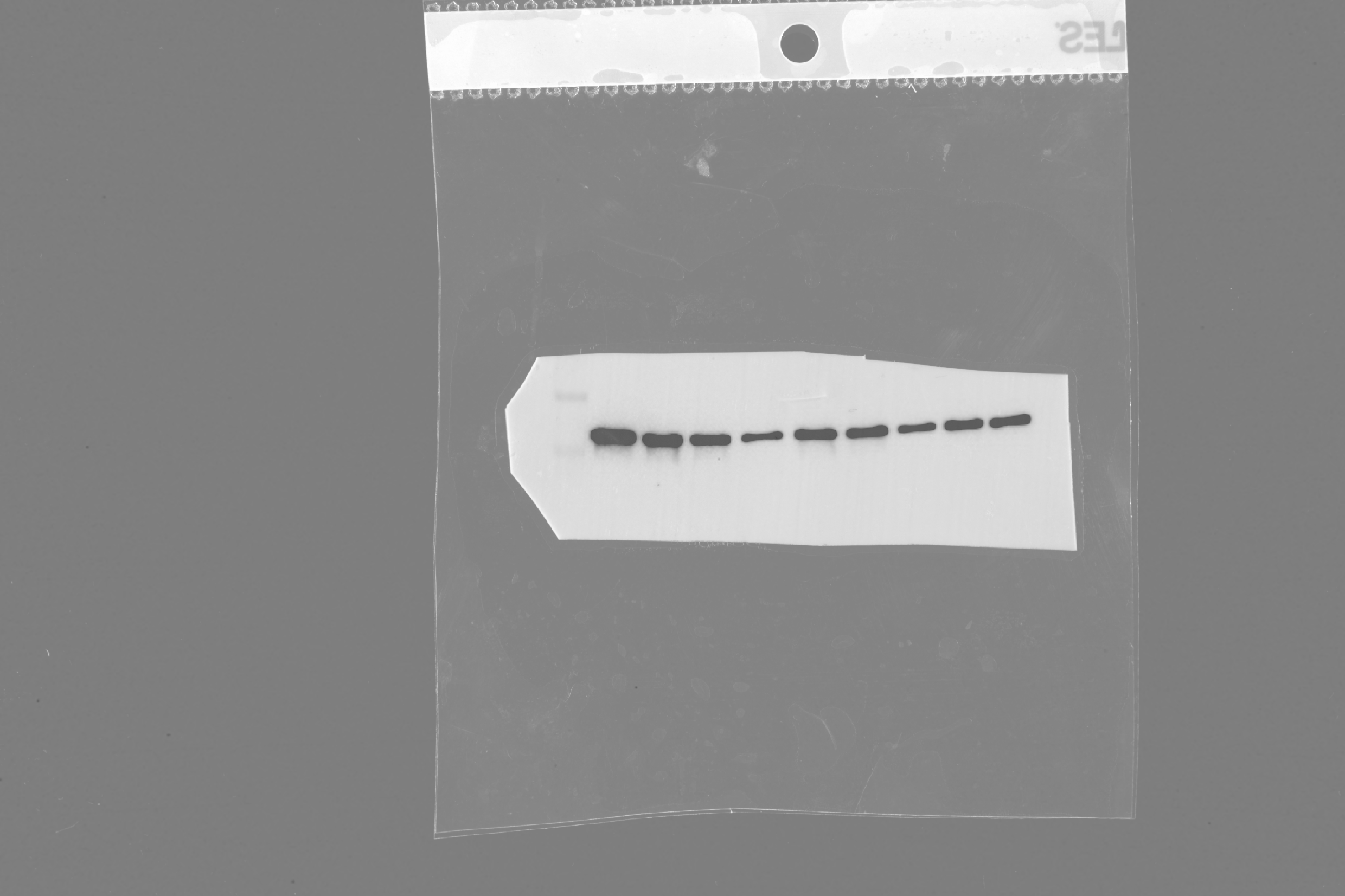

Supplement: Figure 3—figure supplement 1—source data 3. [file elife-103073-fig3-figsupp1-data3.zip › PDGFRA/b-actin NP#1 2 3(Composite).tif]

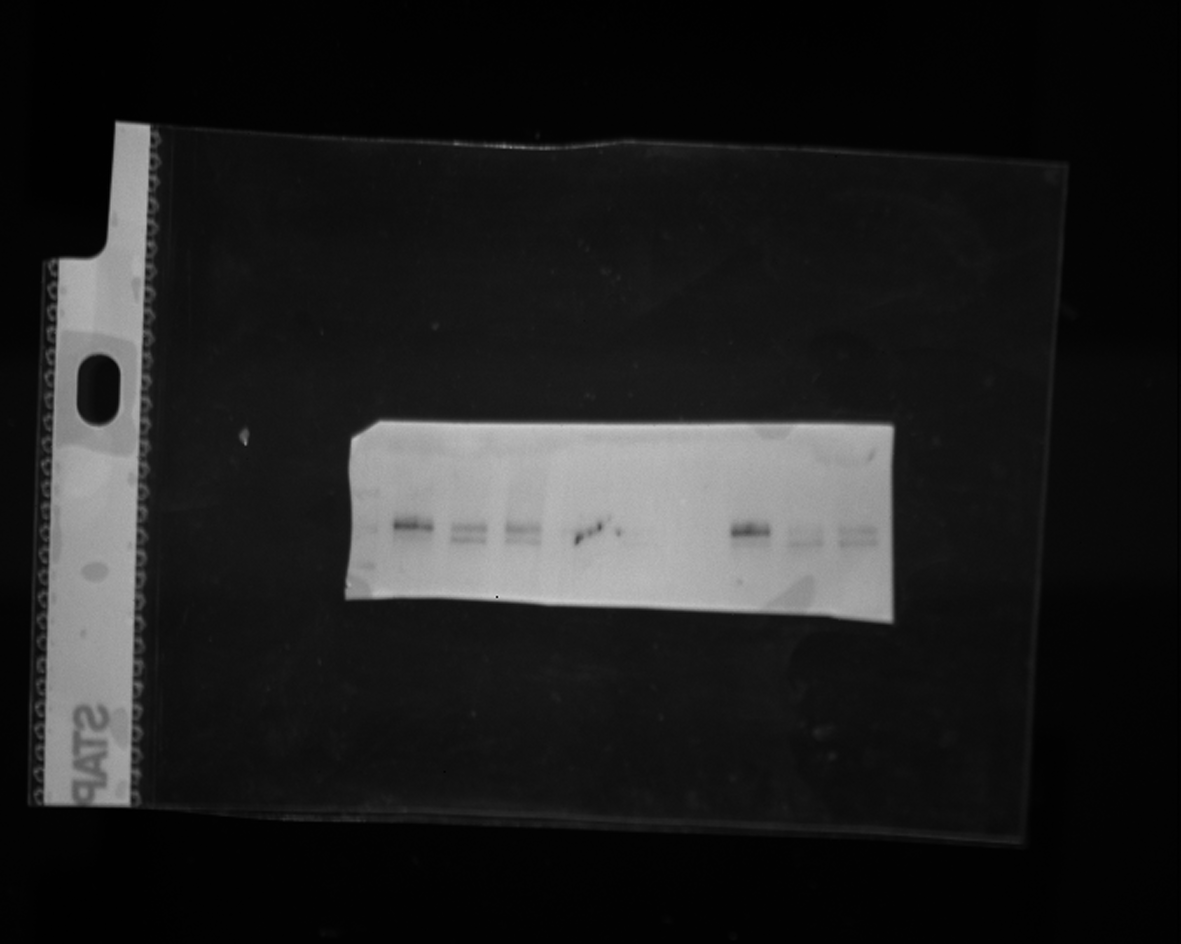

Supplement: Figure 3—figure supplement 1—source data 3. [file elife-103073-fig3-figsupp1-data3.zip › PDGFRA/PDGFRA AF#1 2 3(Overlay).tif]

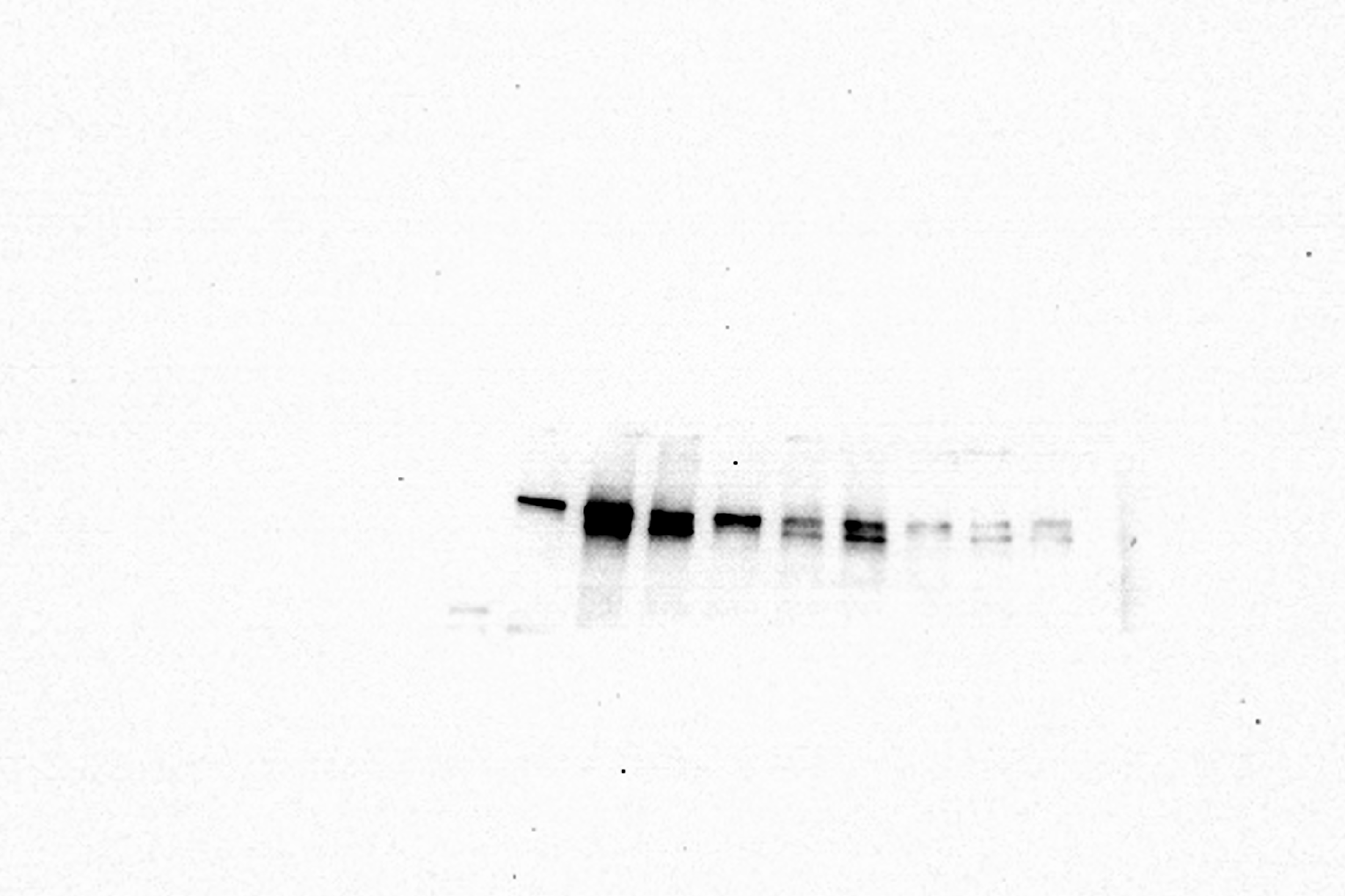

Supplement: Figure 3—figure supplement 1—source data 3. [file elife-103073-fig3-figsupp1-data3.zip › PDGFRA/PDGFRA NP#4 5(Chemiluminescence).tif]

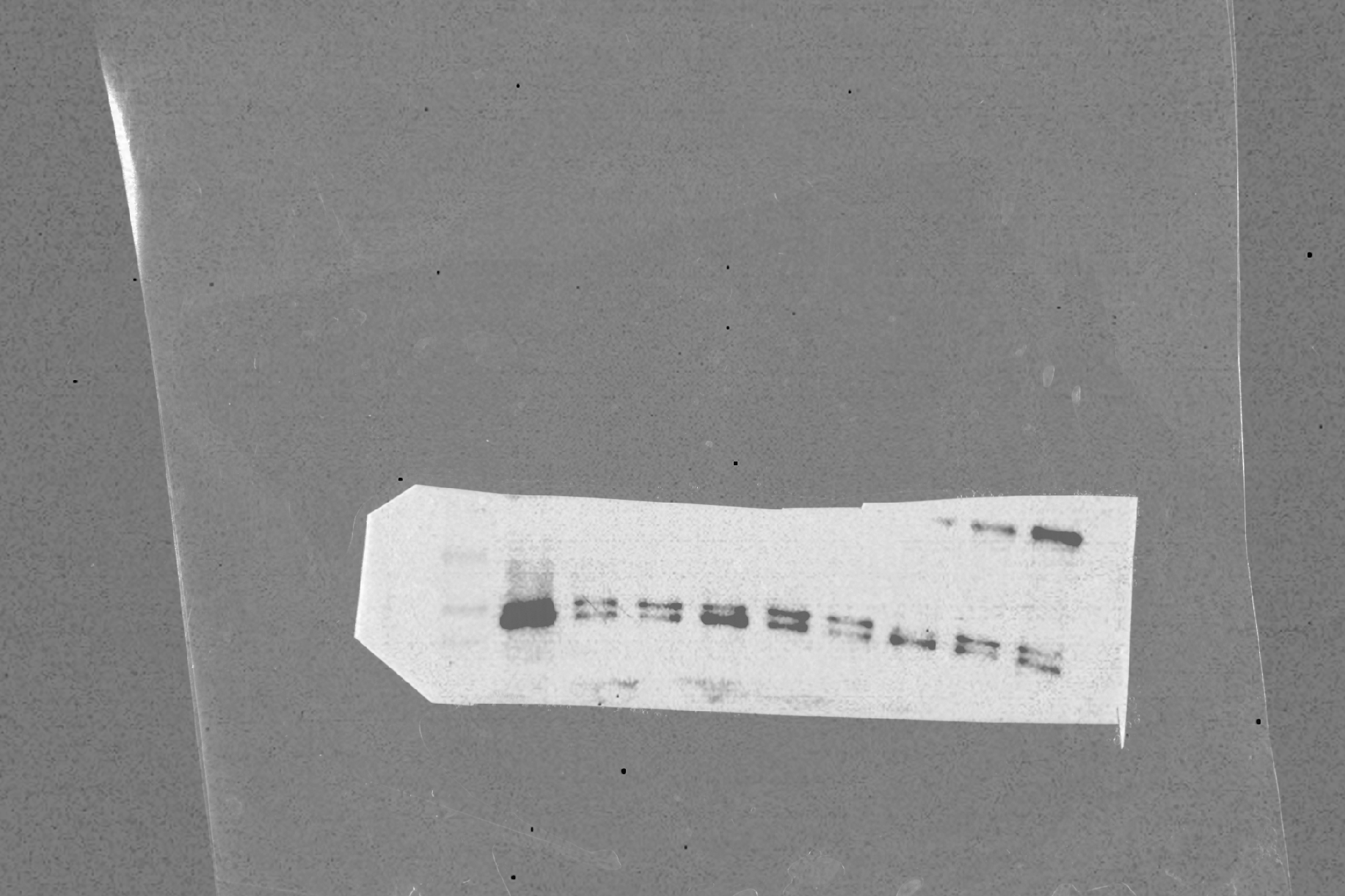

Supplement: Figure 3—figure supplement 1—source data 3. [file elife-103073-fig3-figsupp1-data3.zip › PDGFRA/PDGFRA NP#1 2 3(Composite).tif]

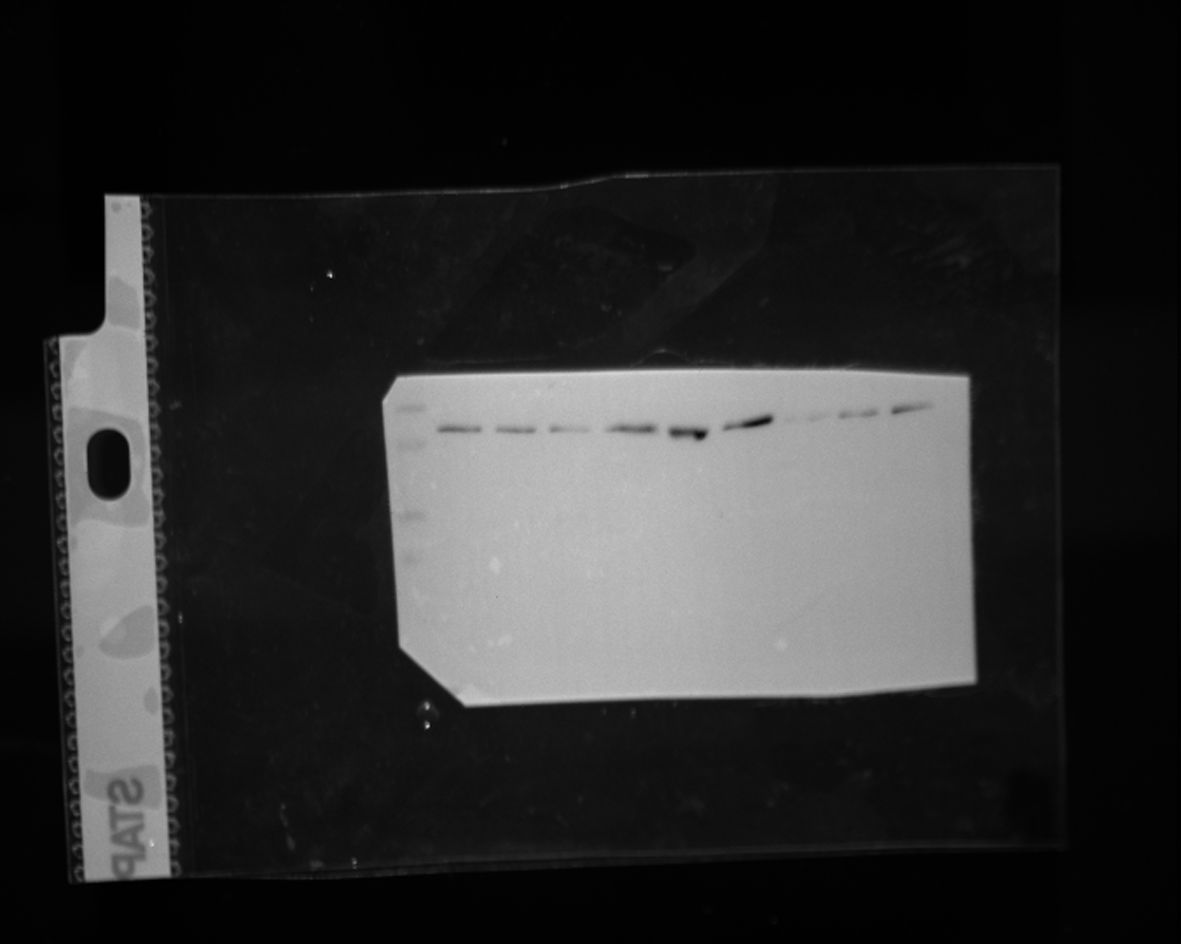

Supplement: Figure 3—figure supplement 1—source data 3. [file elife-103073-fig3-figsupp1-data3.zip › PDGFRA/b-actin AF#4 5 6(Overlay).tif]

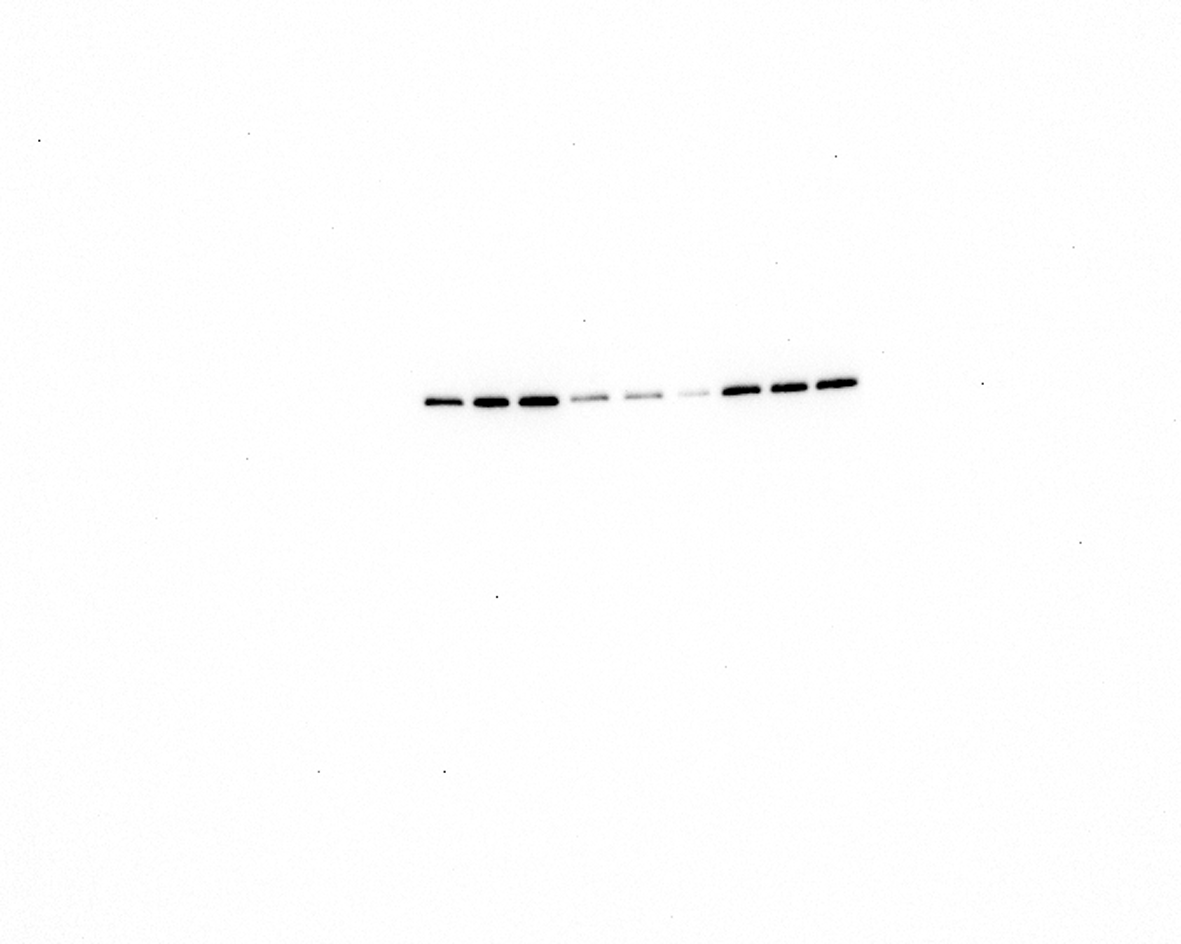

Supplement: Figure 3—figure supplement 1—source data 3. [file elife-103073-fig3-figsupp1-data3.zip › PDGFRA/b-actin AF#1 2 3(Chemi).tif]

NP

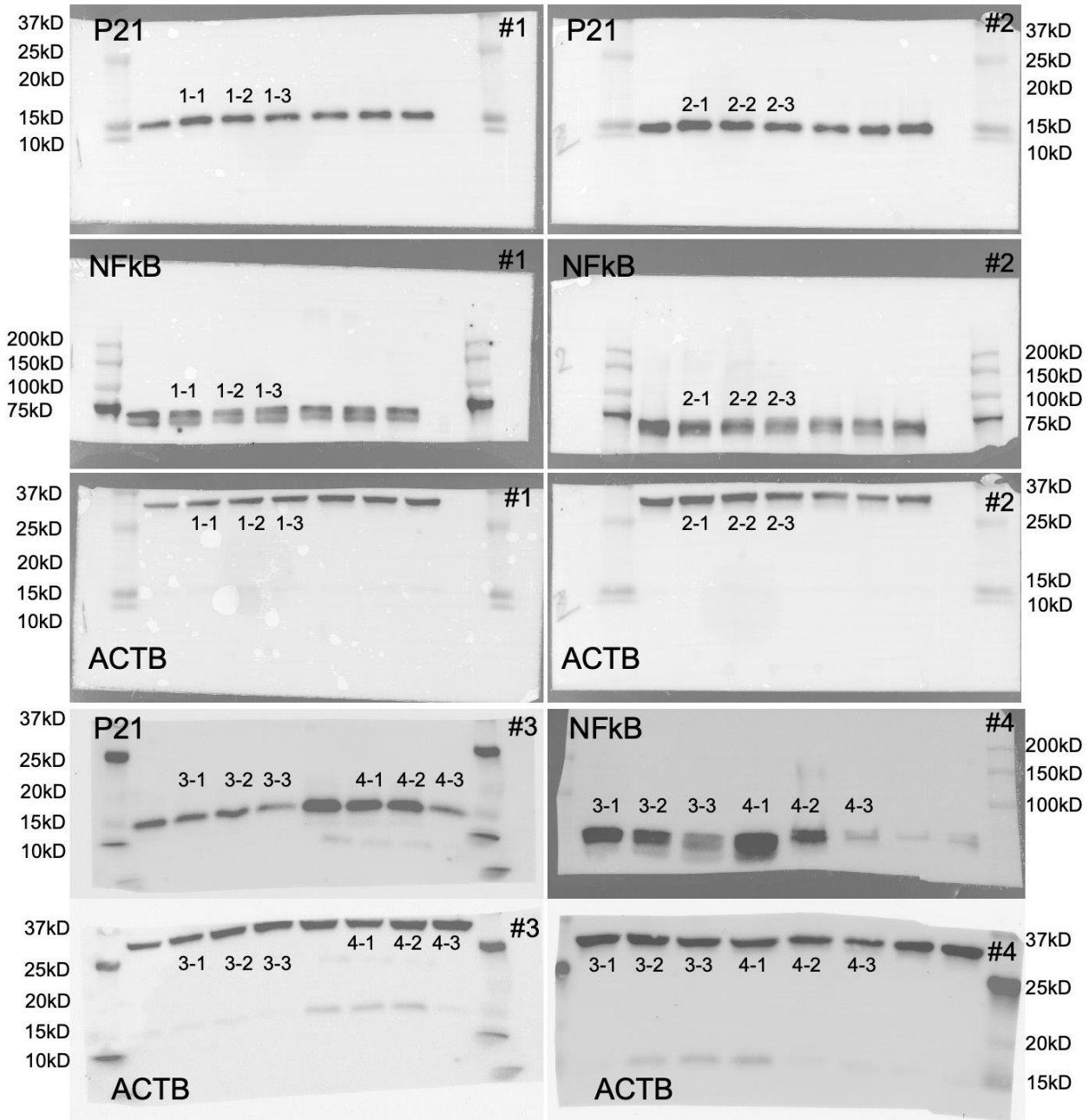

AF

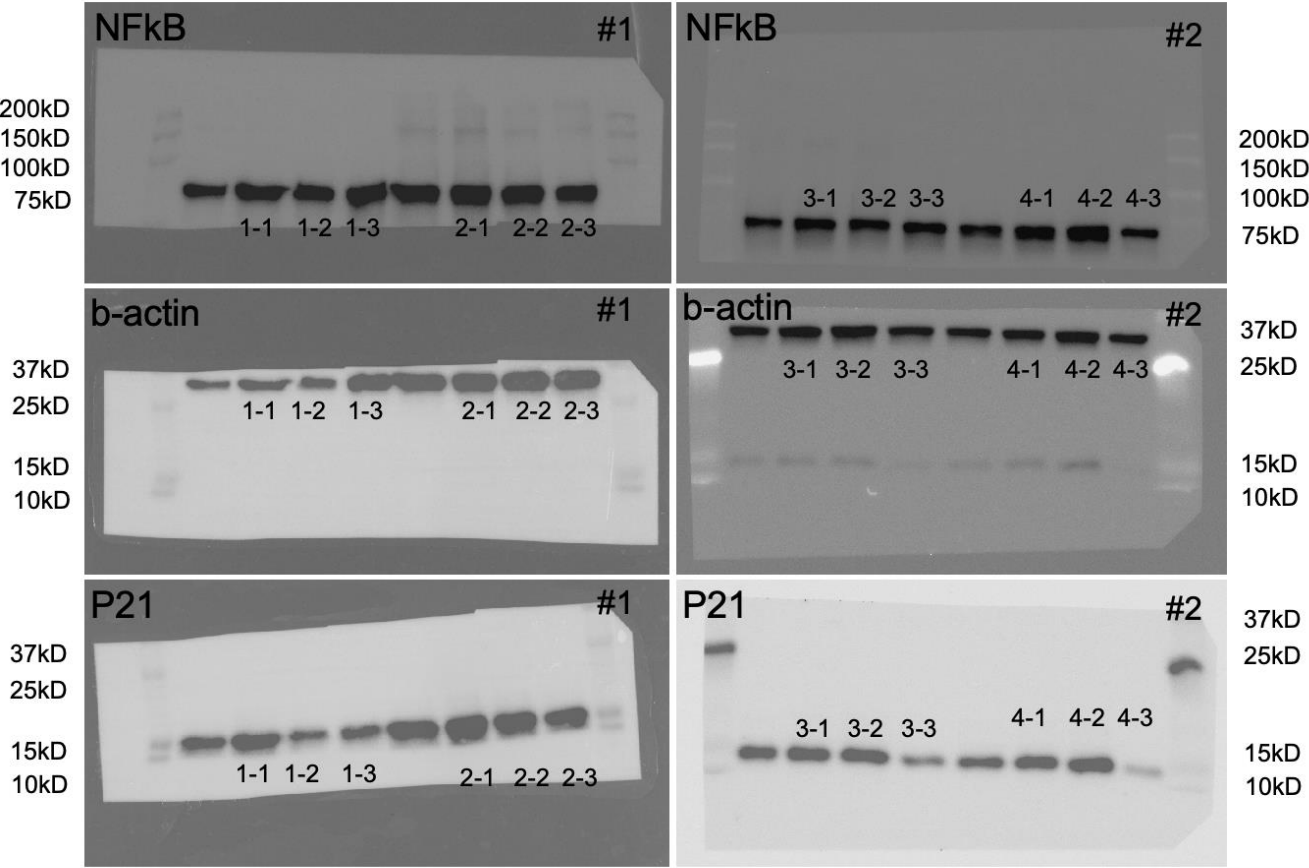

Supplement: Figure 6—source data 2. [file elife-103073-fig6-data2.zip › WB file Figure 6.pdf]

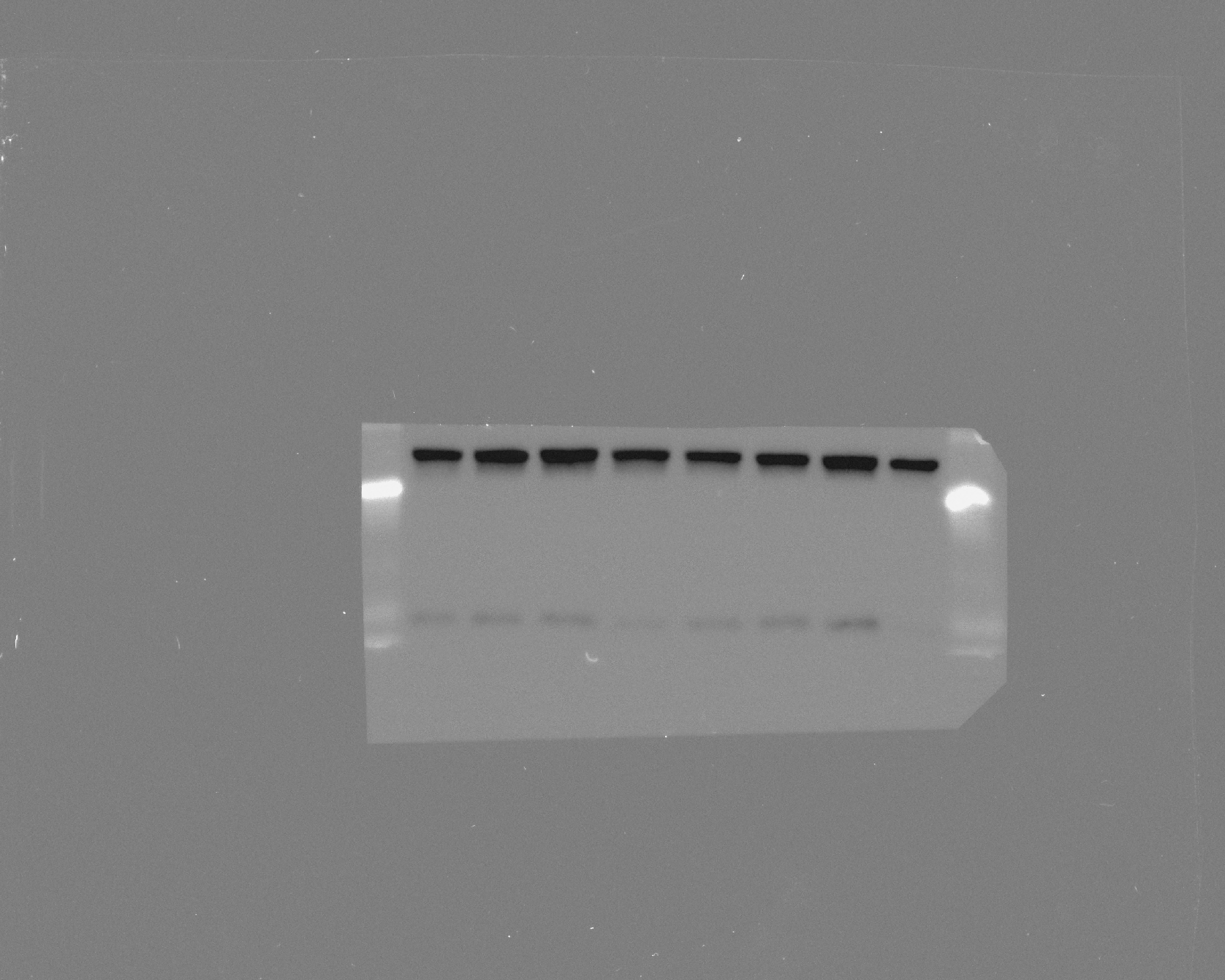

Supplement: Figure 6—source data 3. [file elife-103073-fig6-data3.zip › WB RAW NP AF/AF beta-act #2(Composite).tif]

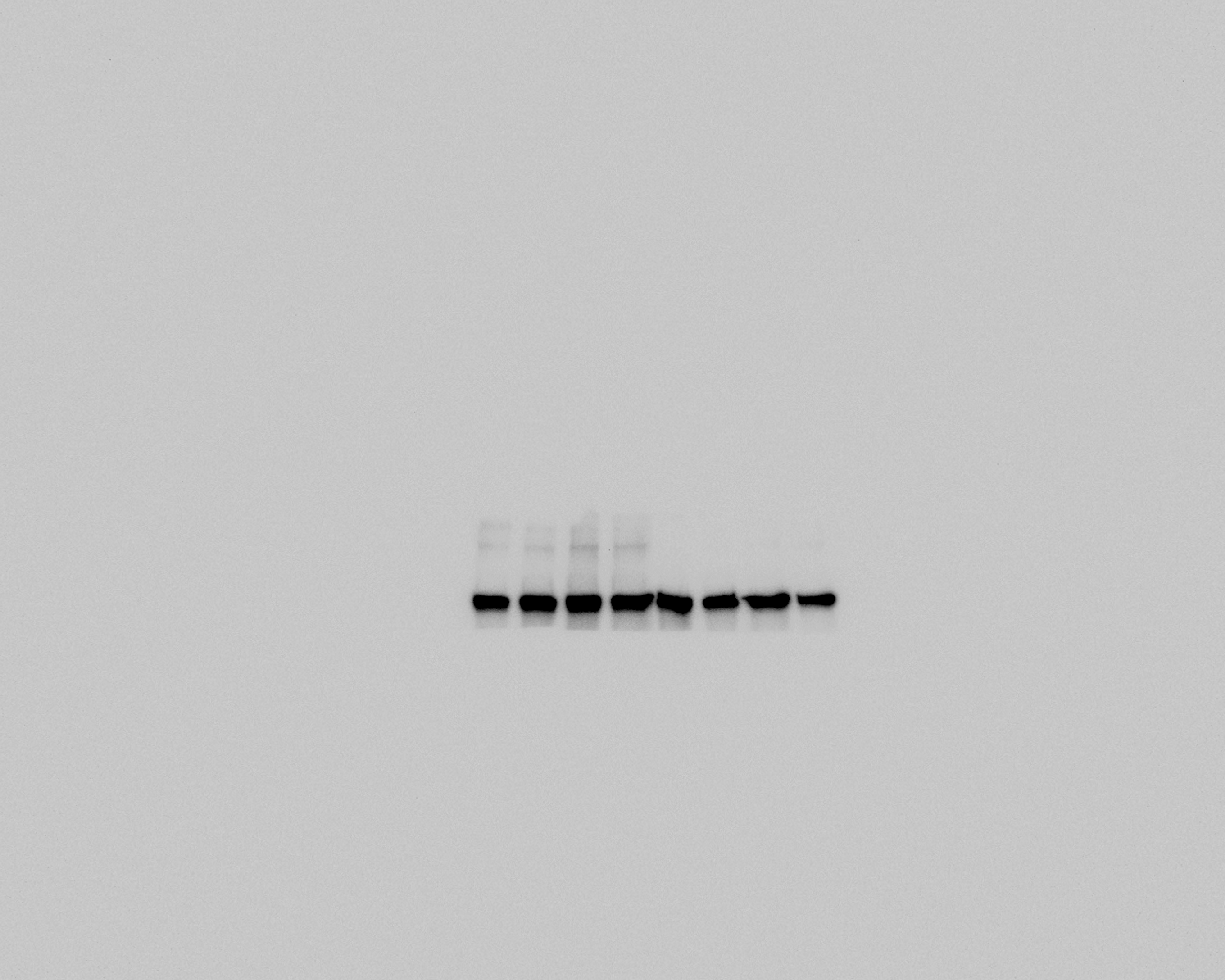

Supplement: Figure 6—source data 3. [file elife-103073-fig6-data3.zip › WB RAW NP AF/AF nfkb #1(Chemiluminescence).tif]

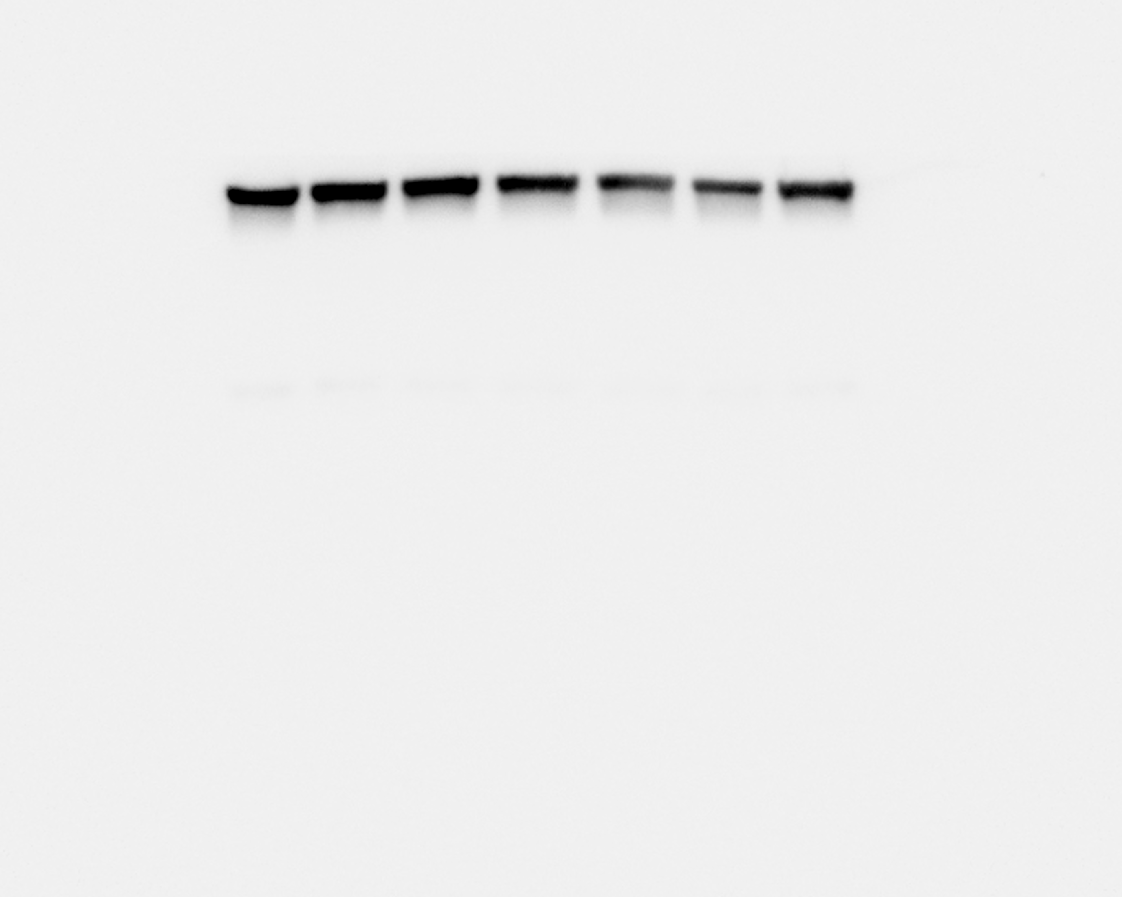

Supplement: Figure 6—source data 3. [file elife-103073-fig6-data3.zip › WB RAW NP AF/NP beta-actin #2(Chemiluminescence).tif]

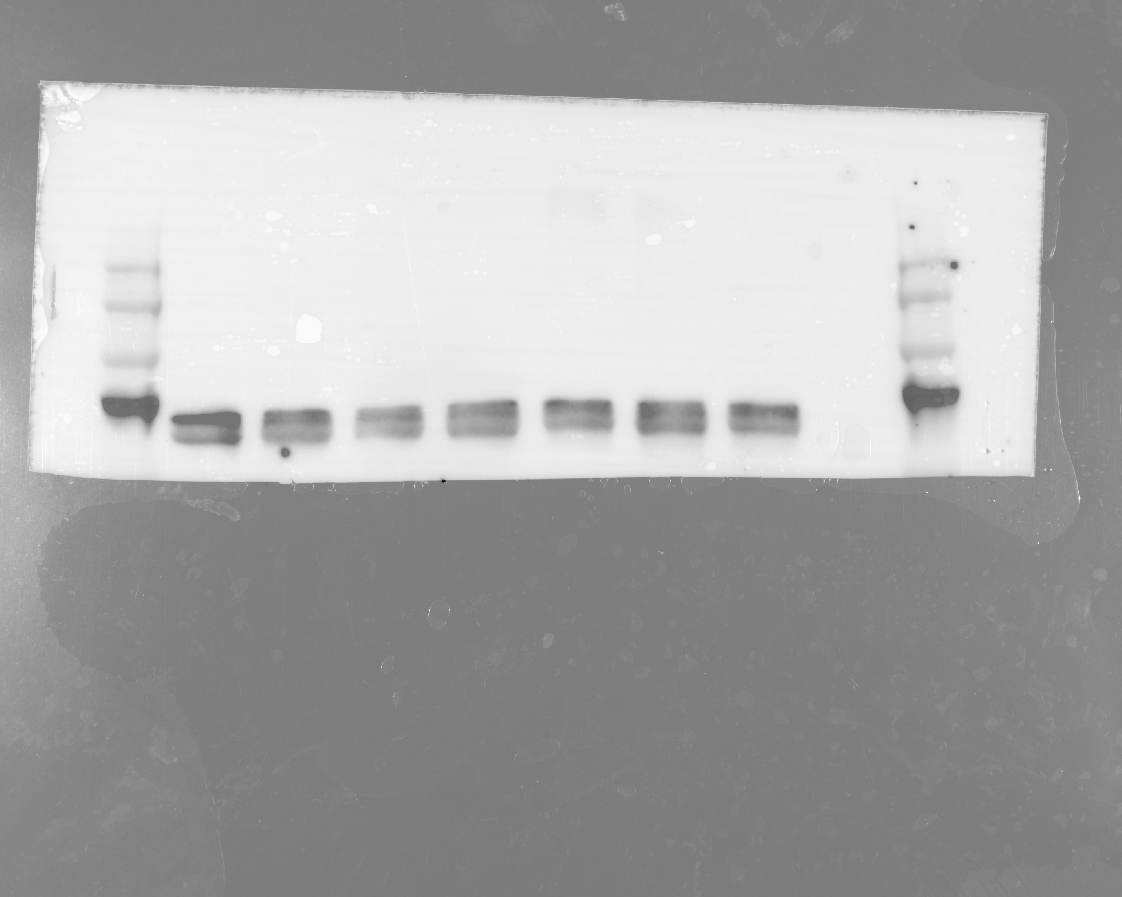

Supplement: Figure 6—source data 3. [file elife-103073-fig6-data3.zip › WB RAW NP AF/NP NFkb #1(Composite).tif]

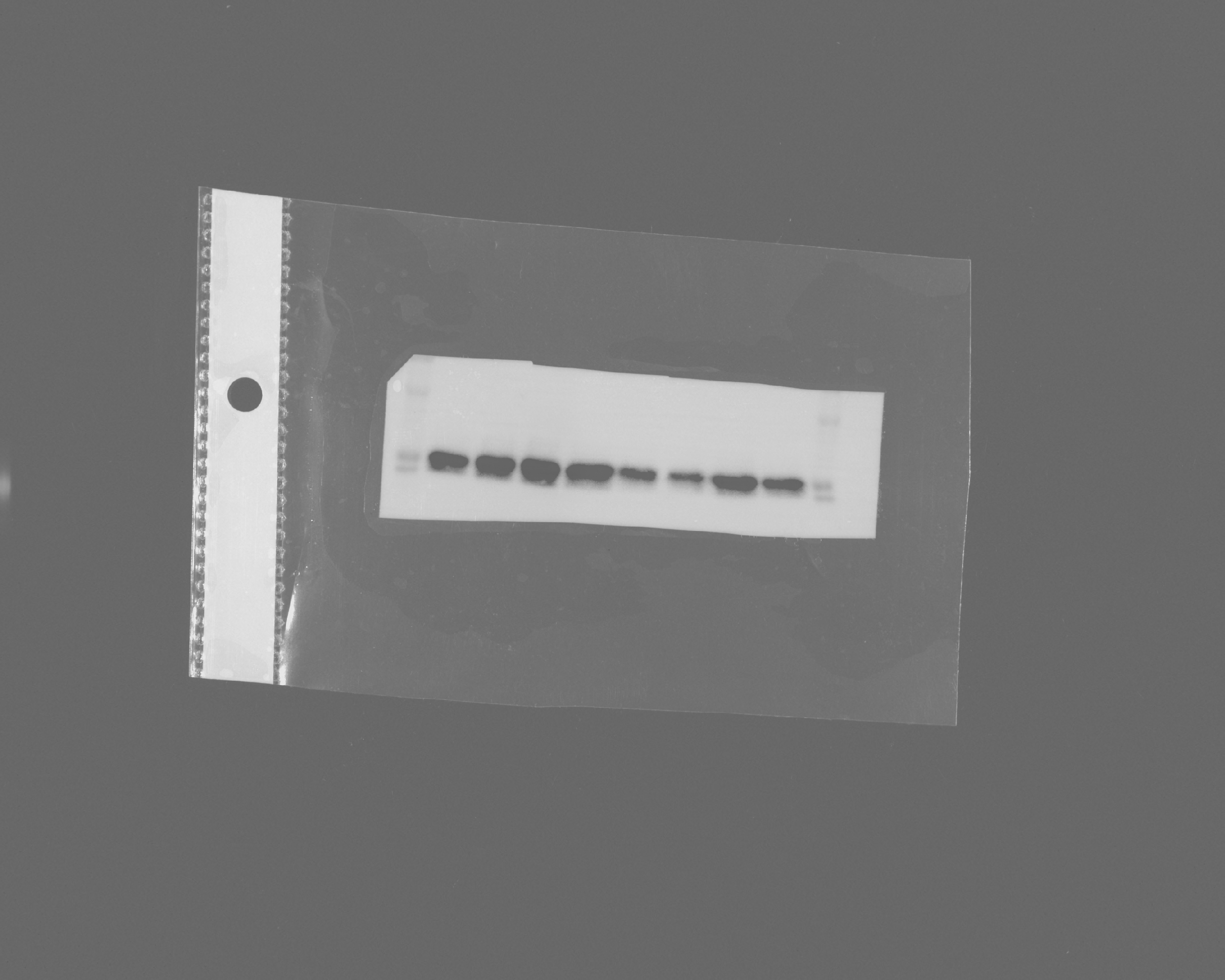

Supplement: Figure 6—source data 3. [file elife-103073-fig6-data3.zip › WB RAW NP AF/AF p21 #1(Composite).tif]

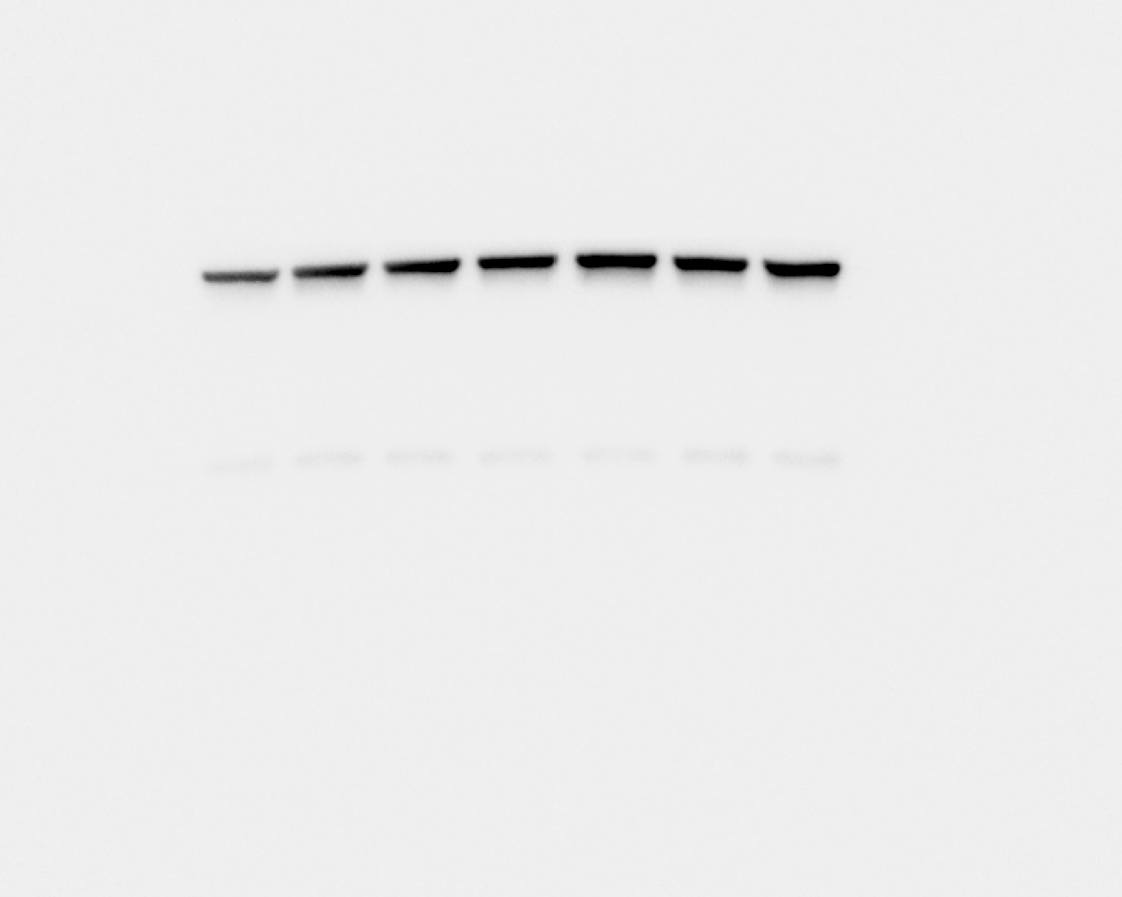

Supplement: Figure 6—source data 3. [file elife-103073-fig6-data3.zip › WB RAW NP AF/NP beta-actin #1(Chemiluminescence).tif]

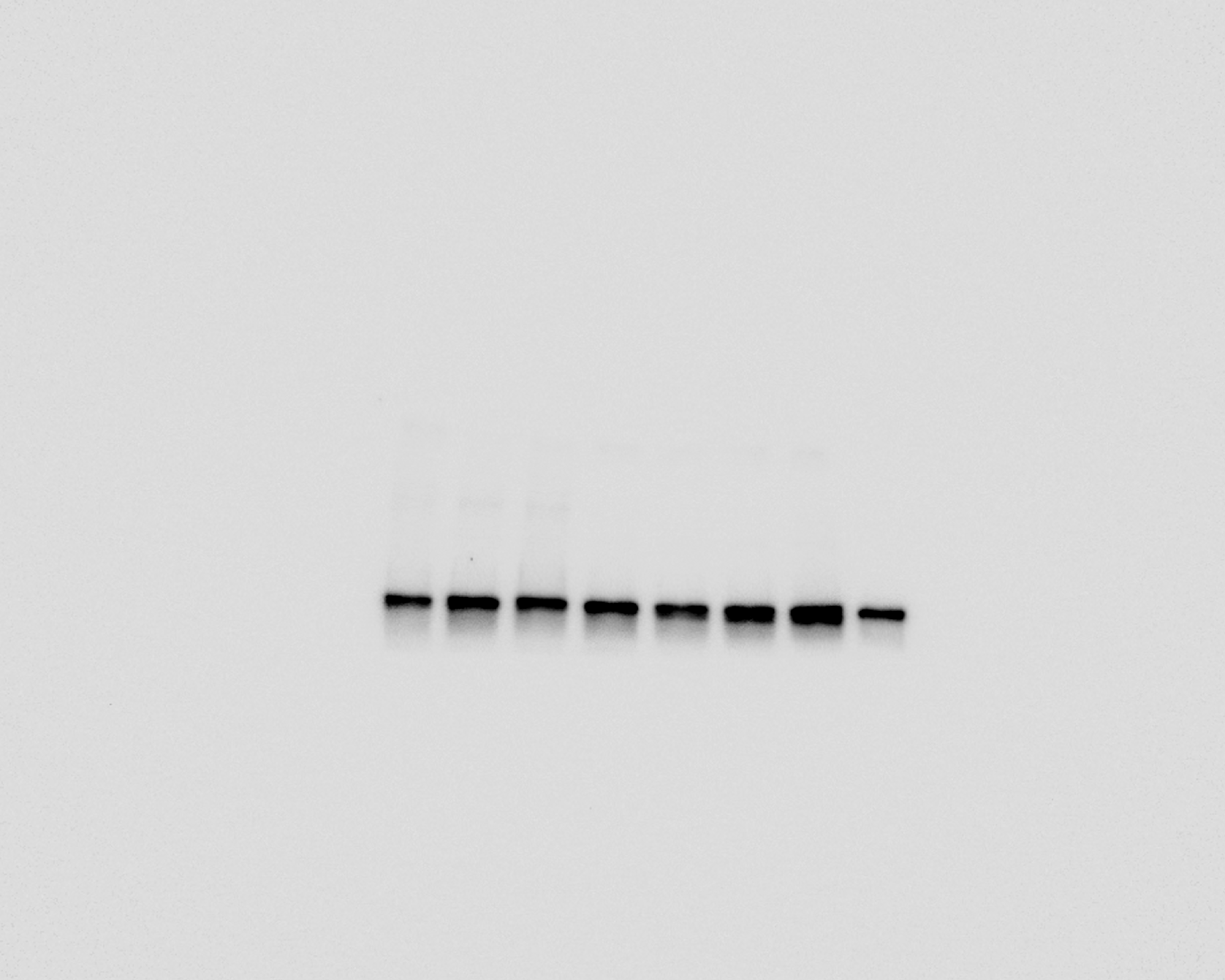

Supplement: Figure 6—source data 3. [file elife-103073-fig6-data3.zip › WB RAW NP AF/AF nfkb #2(Chemiluminescence).tif]

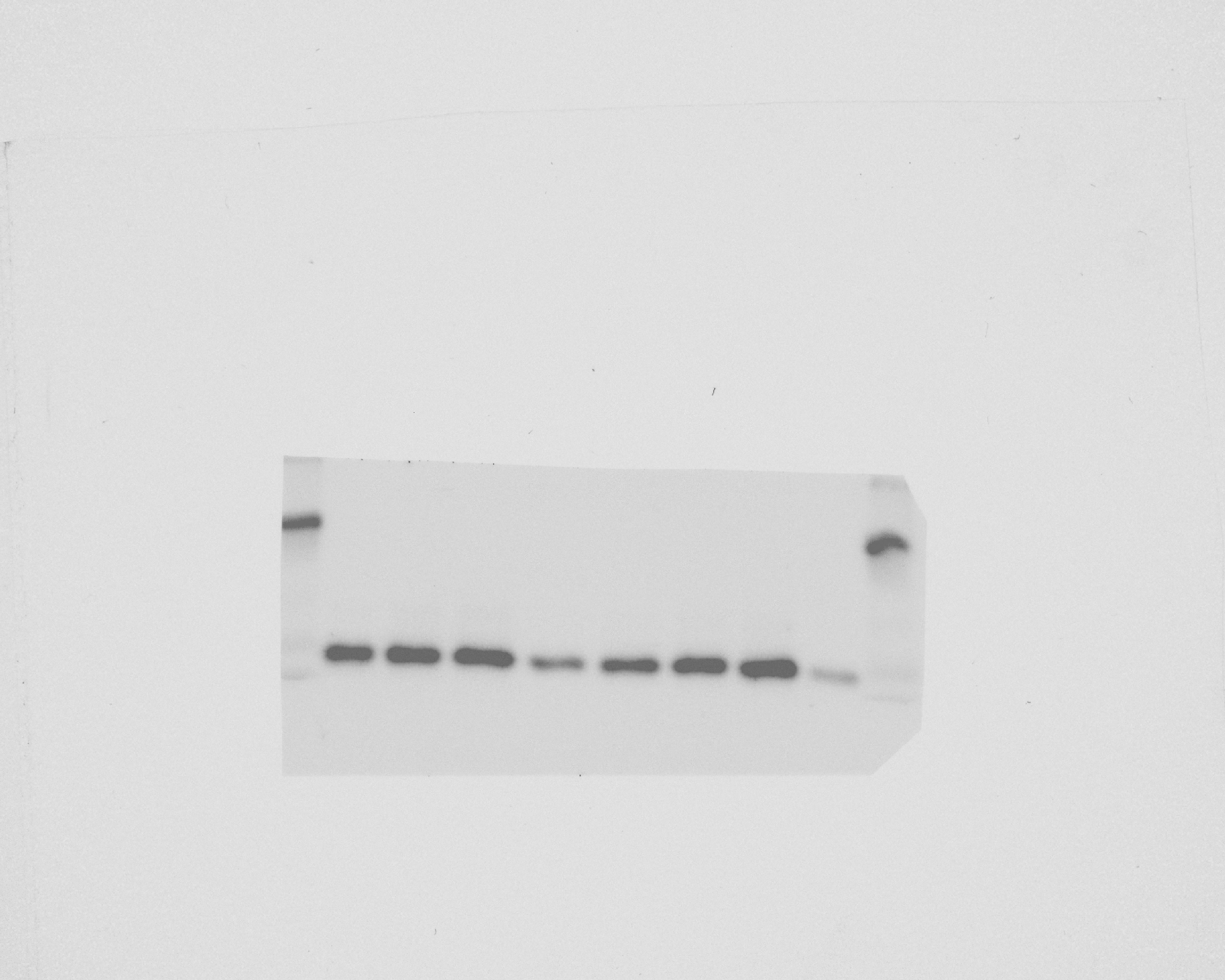

Supplement: Figure 6—source data 3. [file elife-103073-fig6-data3.zip › WB RAW NP AF/AF p21 #2(Composite).tif]

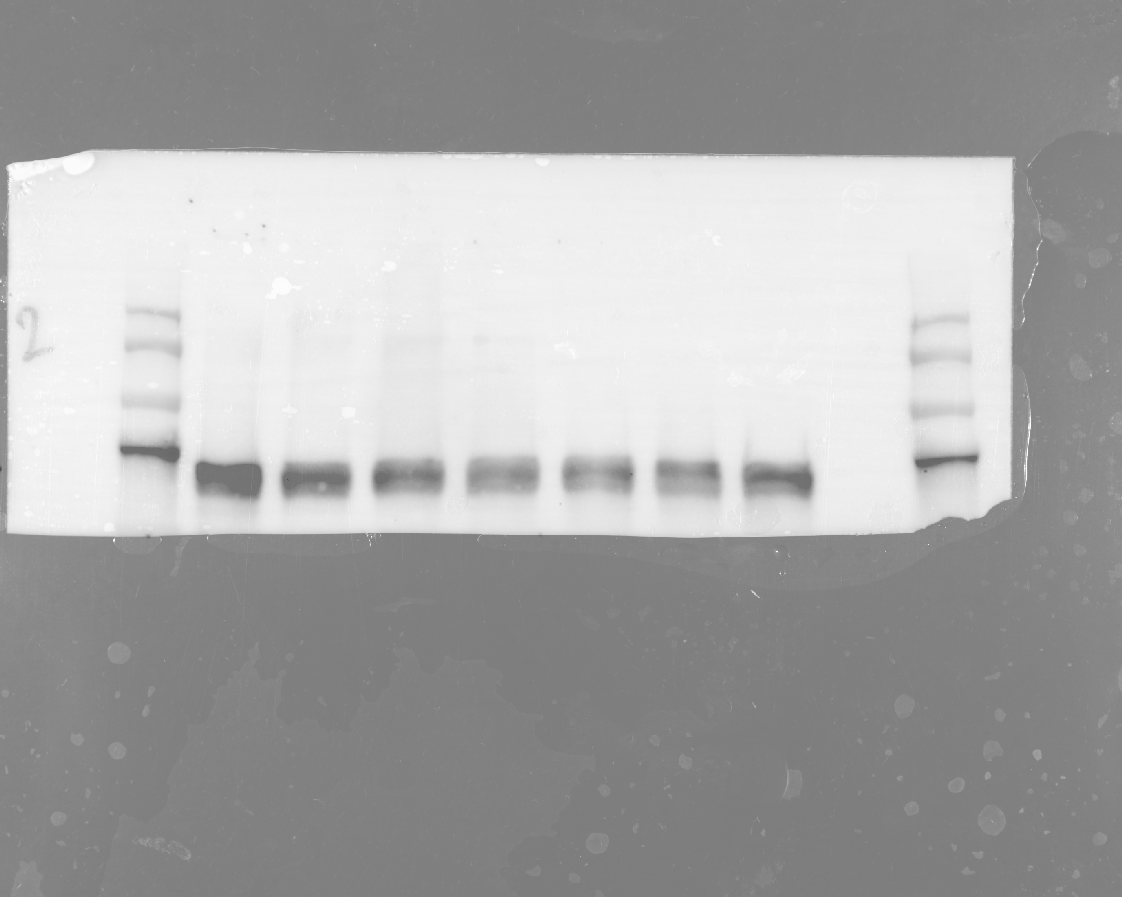

Supplement: Figure 6—source data 3. [file elife-103073-fig6-data3.zip › WB RAW NP AF/NP NFkb #2(Composite).tif]

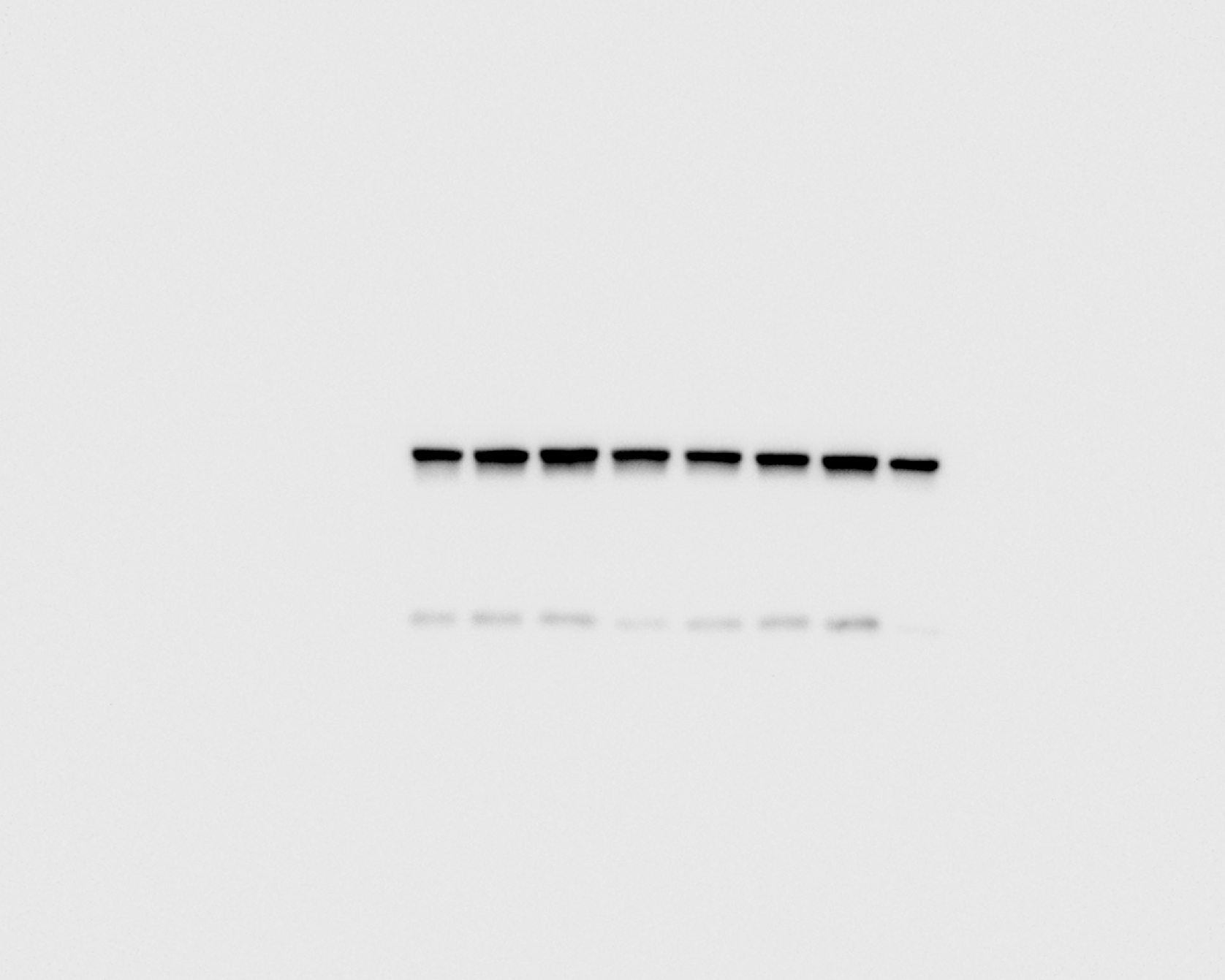

Supplement: Figure 6—source data 3. [file elife-103073-fig6-data3.zip › WB RAW NP AF/AF beta-act #2(Chemiluminescence).tif]

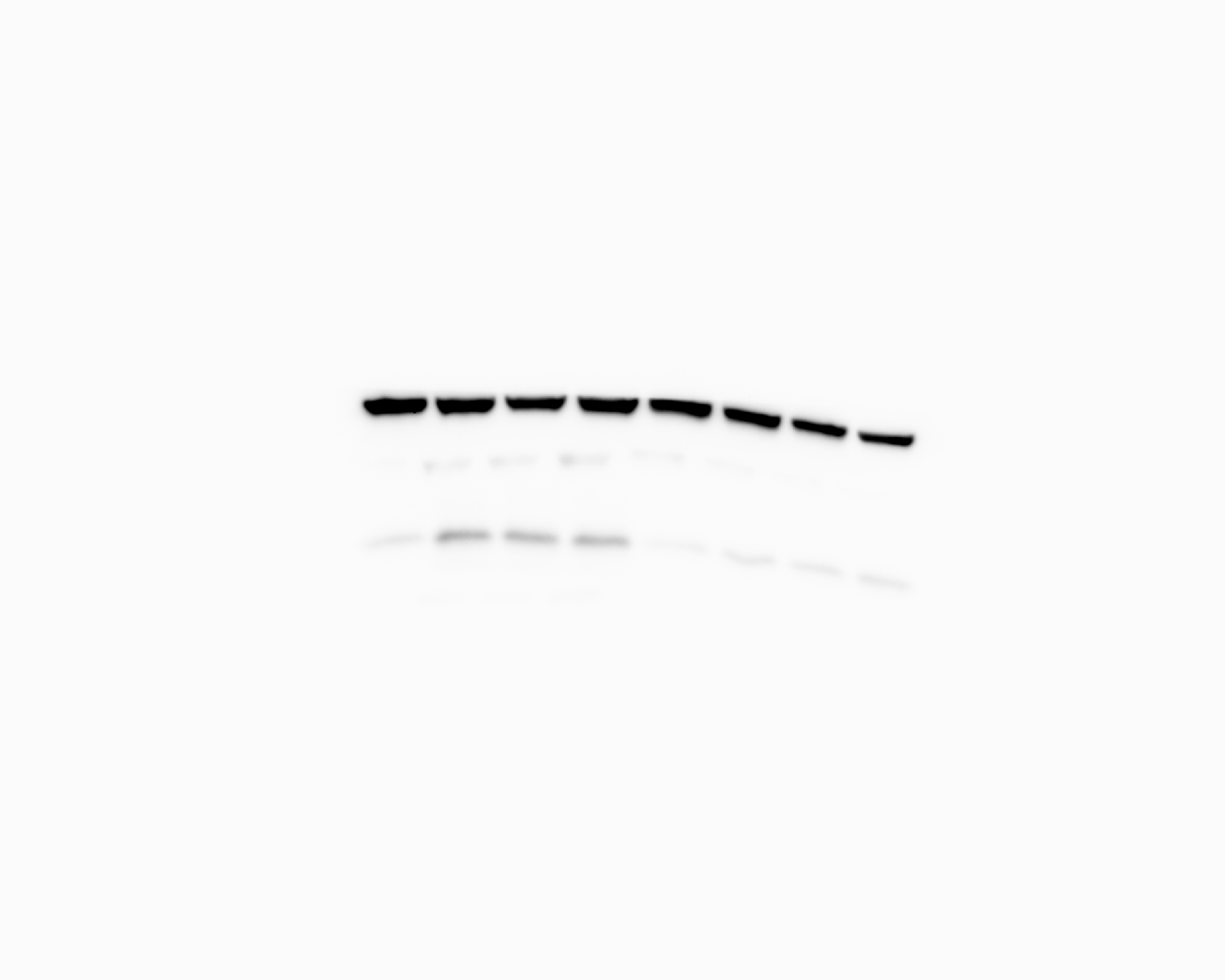

Supplement: Figure 6—source data 3. [file elife-103073-fig6-data3.zip › WB RAW NP AF/NP beta-actin #3(Chemiluminescence).tif]

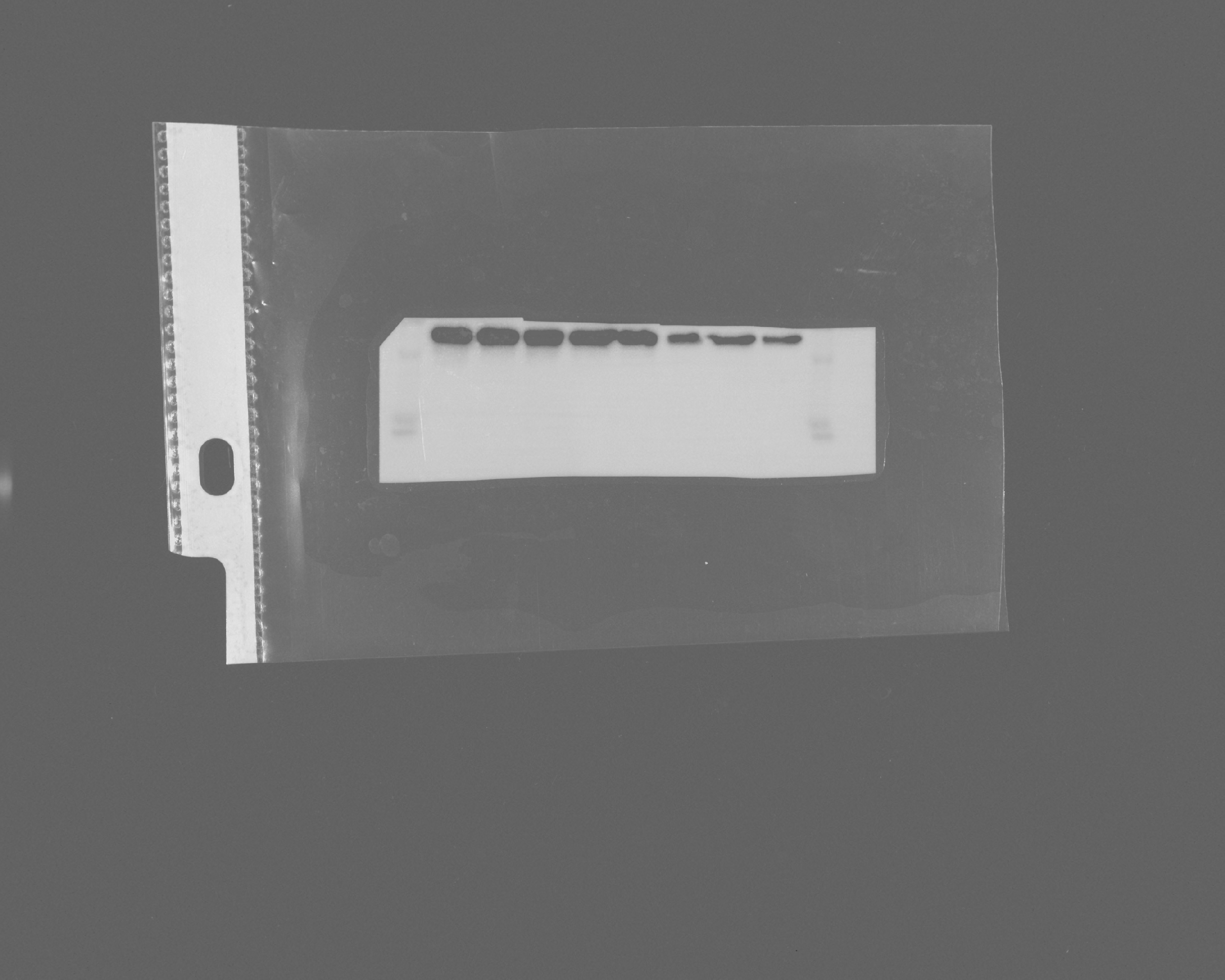

Supplement: Figure 6—source data 3. [file elife-103073-fig6-data3.zip › WB RAW NP AF/AF beta-actin #1(Composite).tif]

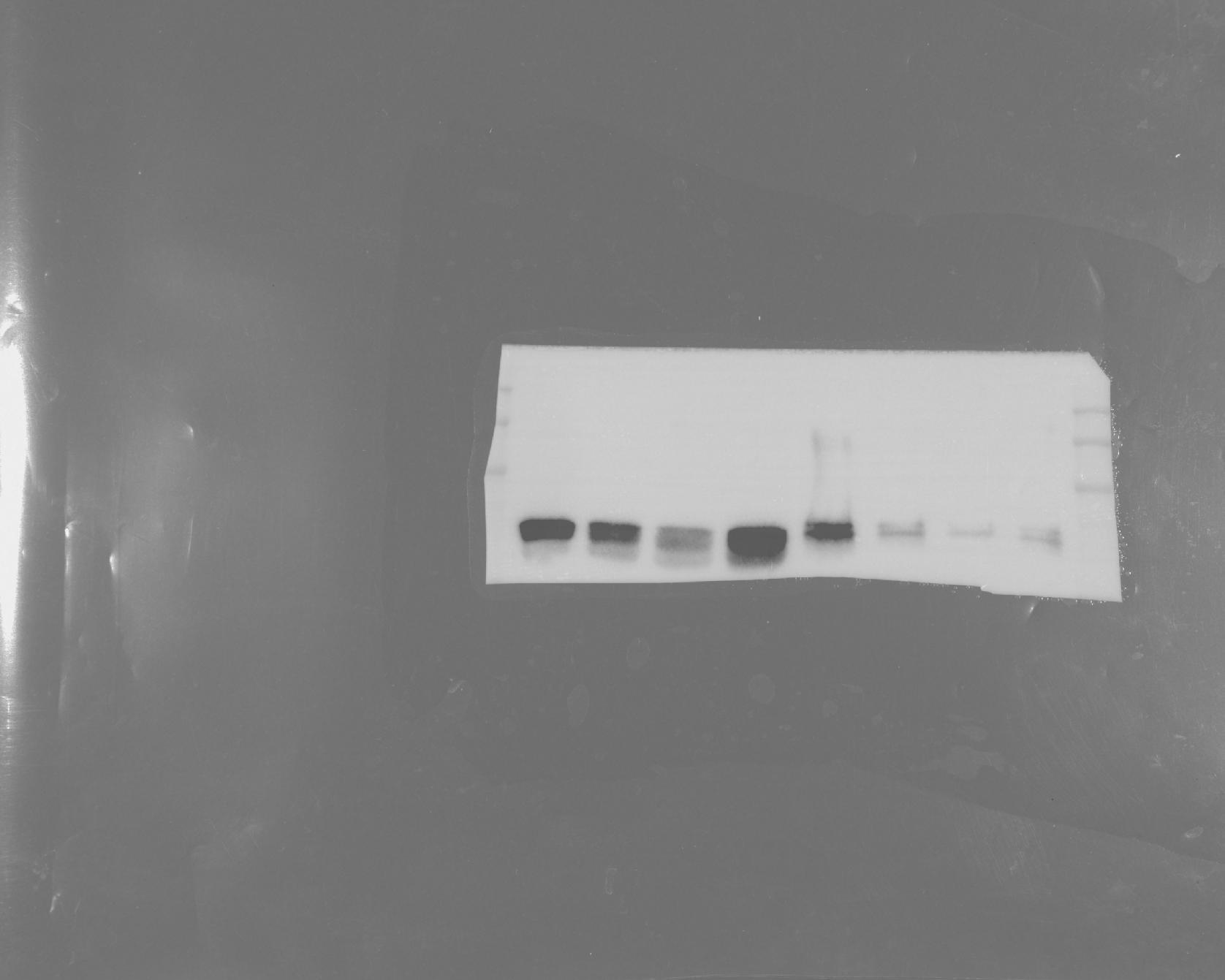

Supplement: Figure 6—source data 3. [file elife-103073-fig6-data3.zip › WB RAW NP AF/NP NFkb #4(Composite).tif]

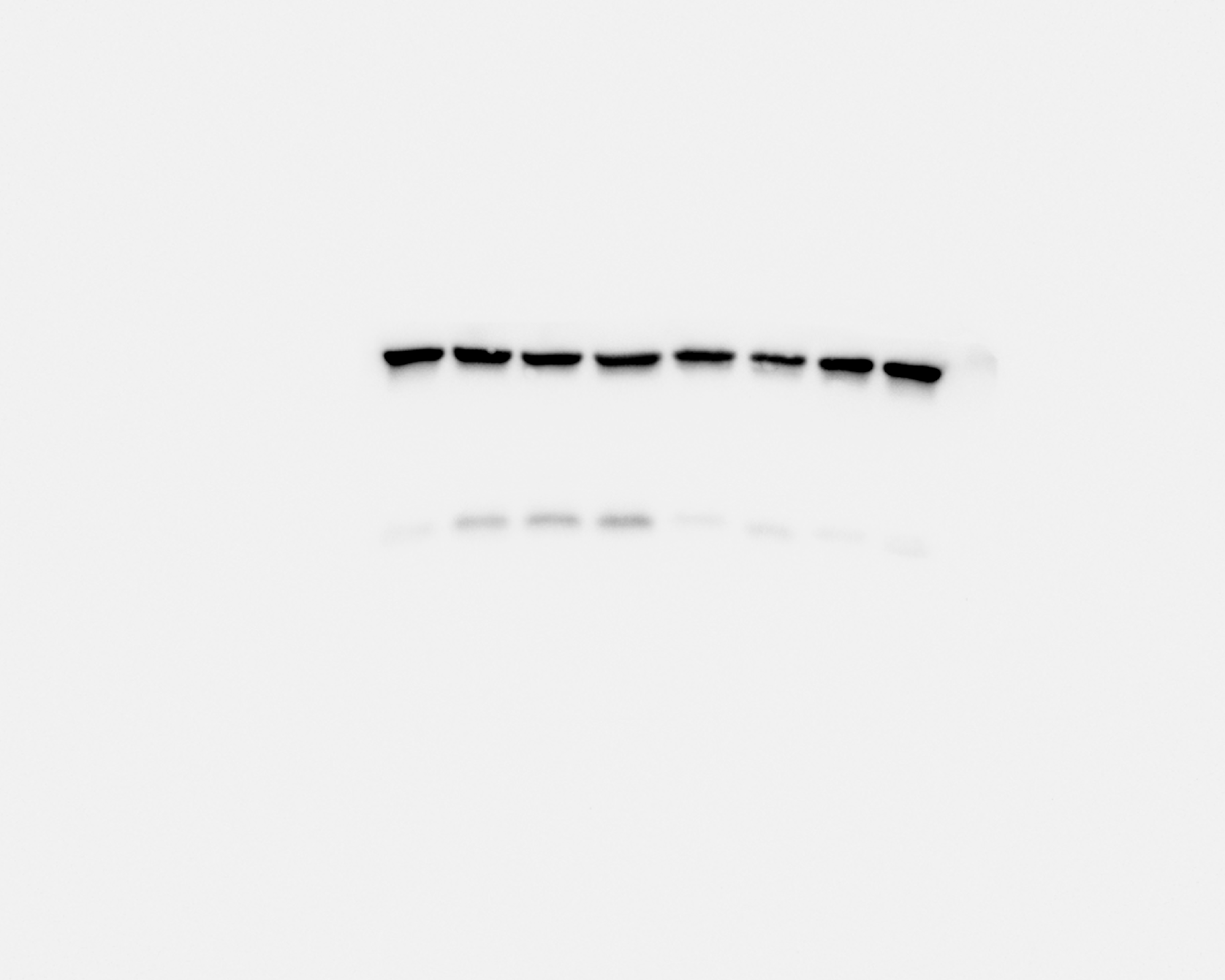

Supplement: Figure 6—source data 3. [file elife-103073-fig6-data3.zip › WB RAW NP AF/NP beta-actin #4(Chemiluminescence).tif]

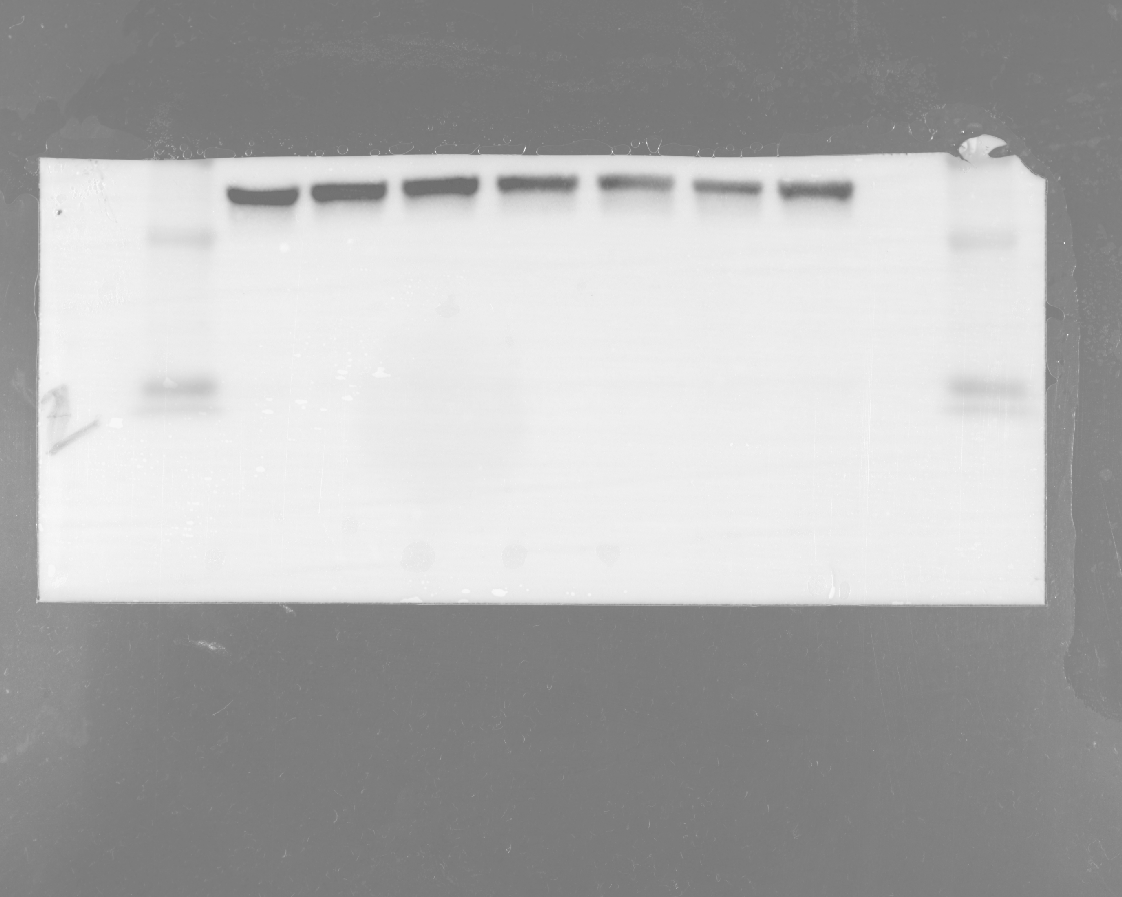

Supplement: Figure 6—source data 3. [file elife-103073-fig6-data3.zip › WB RAW NP AF/NP beta-actin #2(Composite).tif]

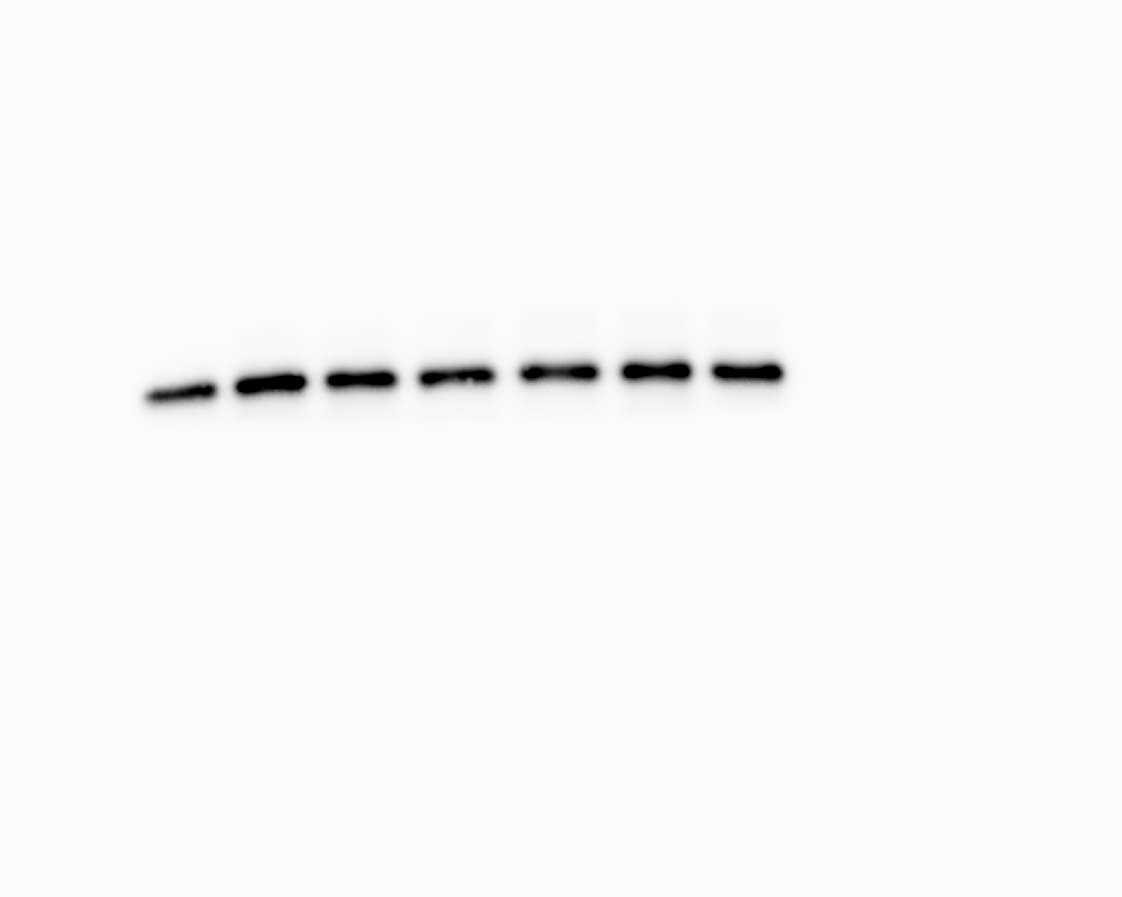

Supplement: Figure 6—source data 3. [file elife-103073-fig6-data3.zip › WB RAW NP AF/NP P21 #1(Chemiluminescence).tif]

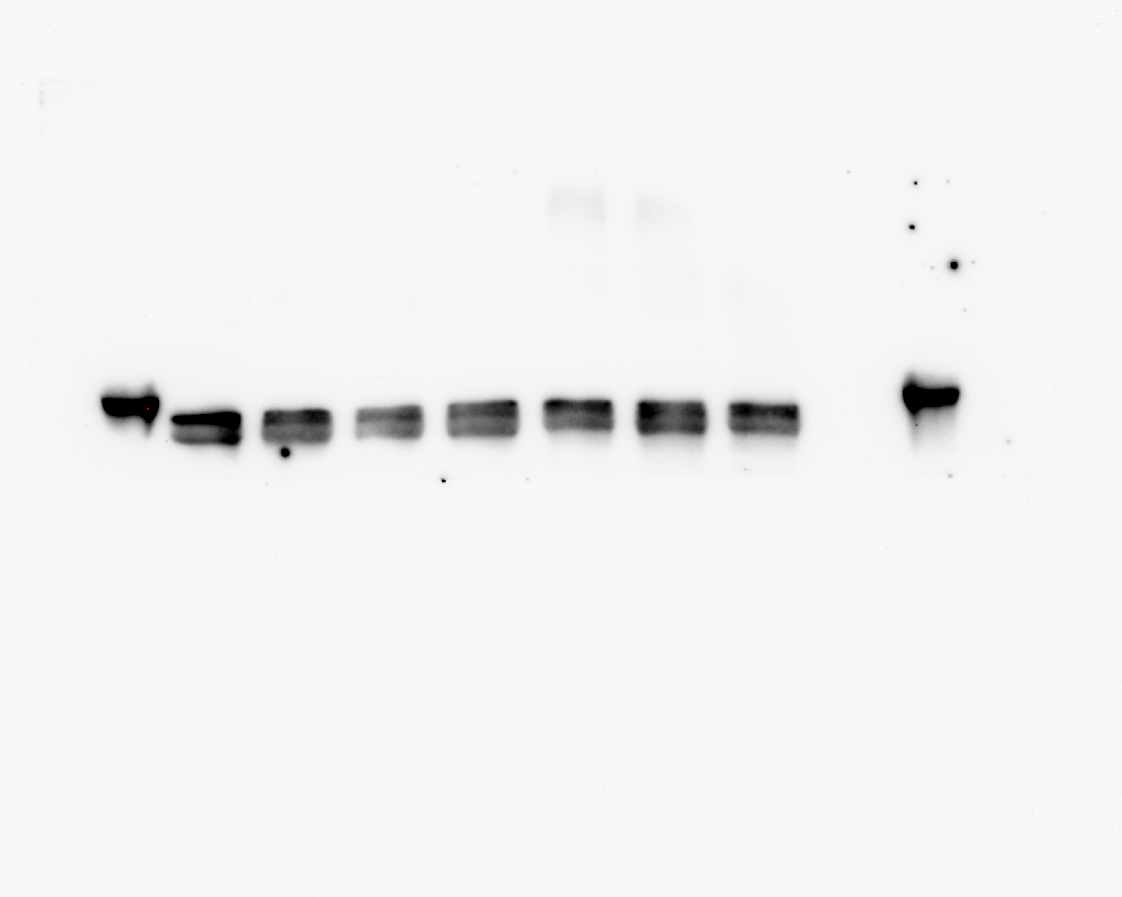

Supplement: Figure 6—source data 3. [file elife-103073-fig6-data3.zip › WB RAW NP AF/NP NFkb #1(Chemiluminescence).tif]

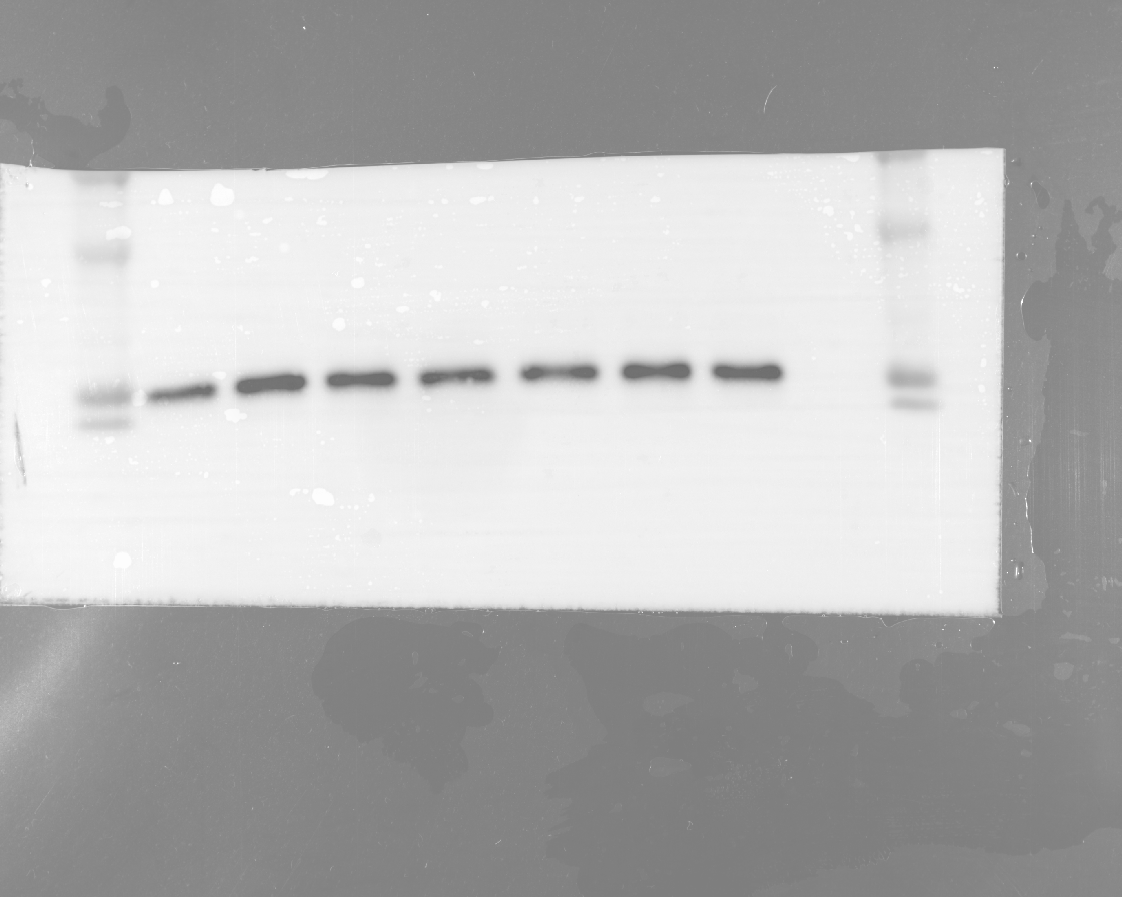

Supplement: Figure 6—source data 3. [file elife-103073-fig6-data3.zip › WB RAW NP AF/NP P21 #1(Composite).tif]

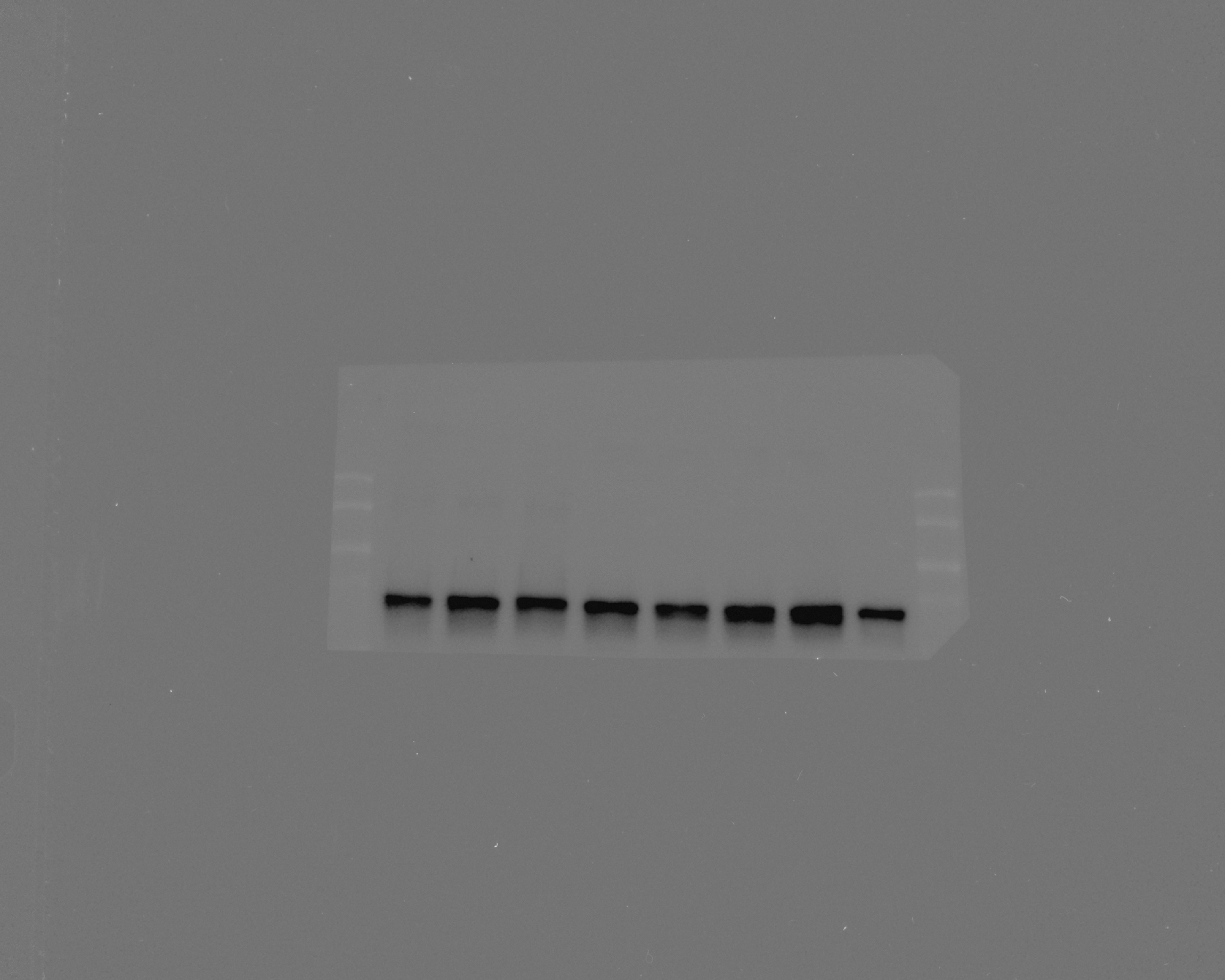

Supplement: Figure 6—source data 3. [file elife-103073-fig6-data3.zip › WB RAW NP AF/AF nfkb #2(Composite).tif]

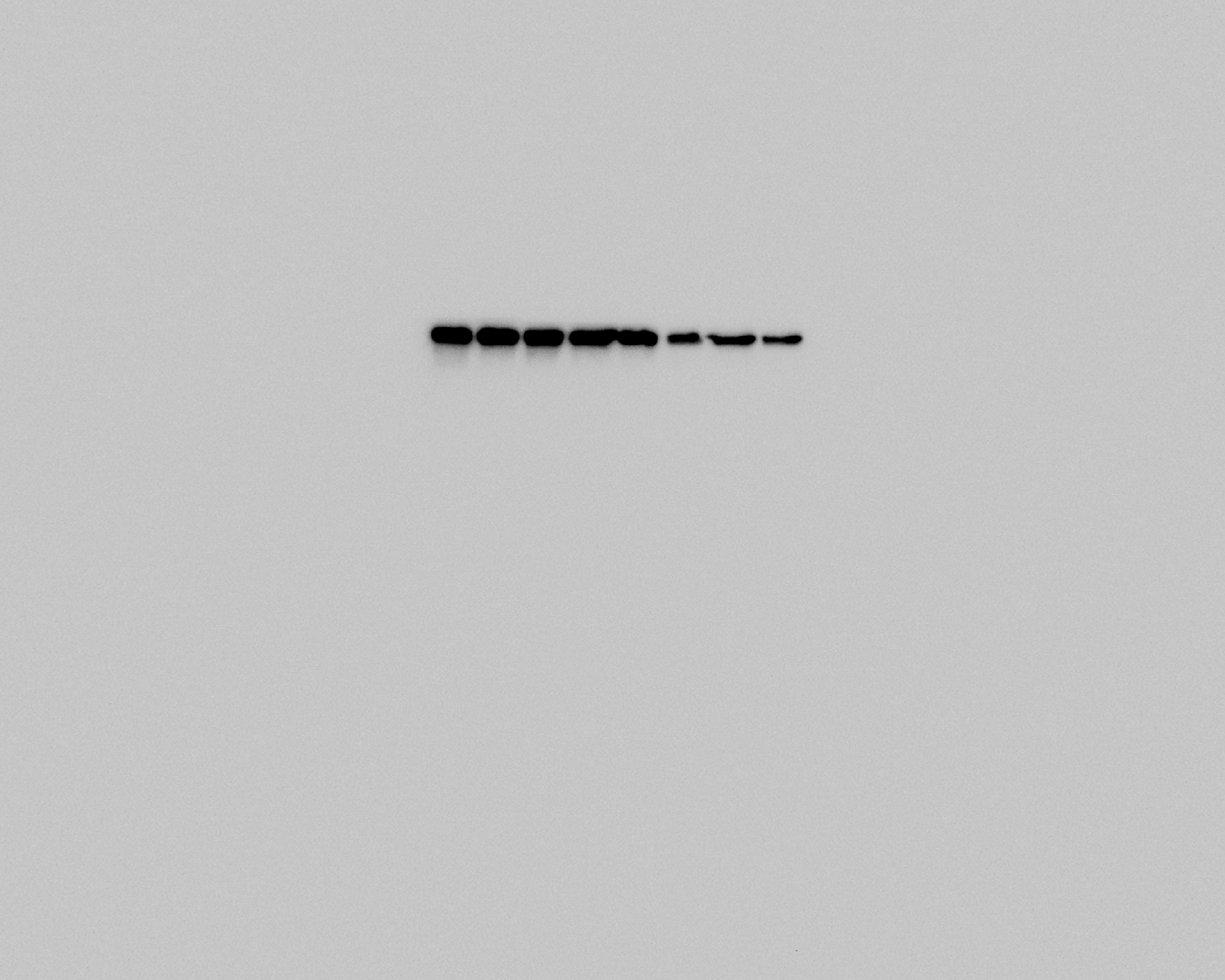

Supplement: Figure 6—source data 3. [file elife-103073-fig6-data3.zip › WB RAW NP AF/AF beta-actin #1(Chemiluminescence).tif]

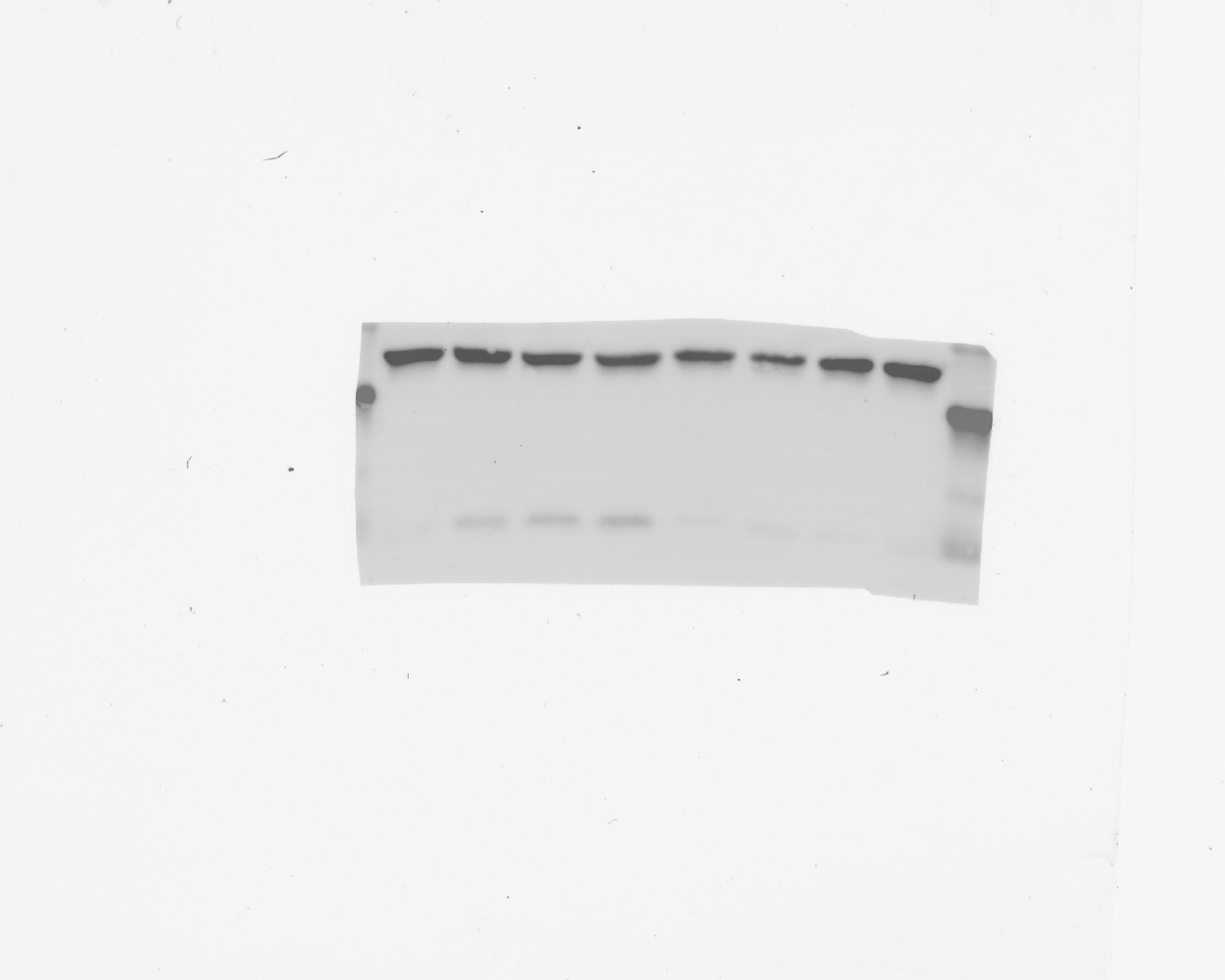

Supplement: Figure 6—source data 3. [file elife-103073-fig6-data3.zip › WB RAW NP AF/NP beta-actin #4(Composite).tif]

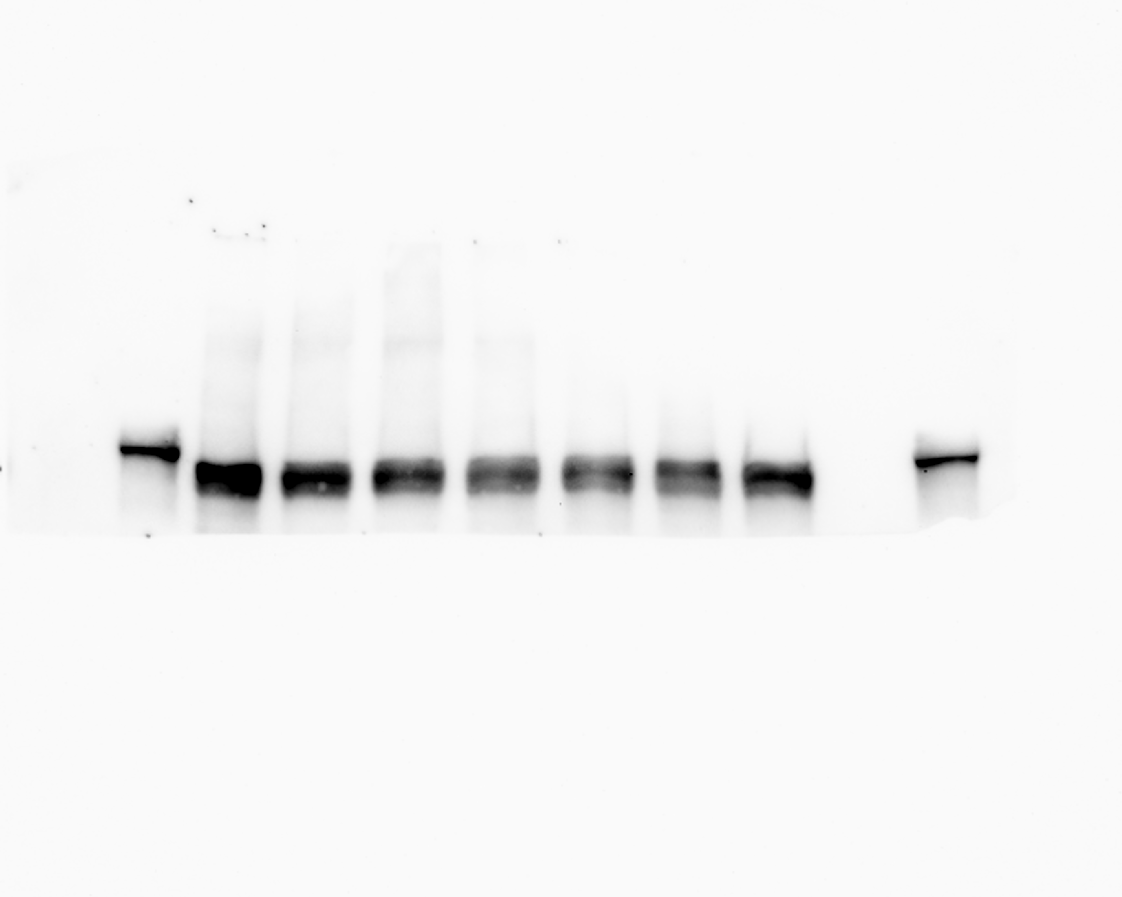

Supplement: Figure 6—source data 3. [file elife-103073-fig6-data3.zip › WB RAW NP AF/NP NFkb #2(Chemiluminescence).tif]

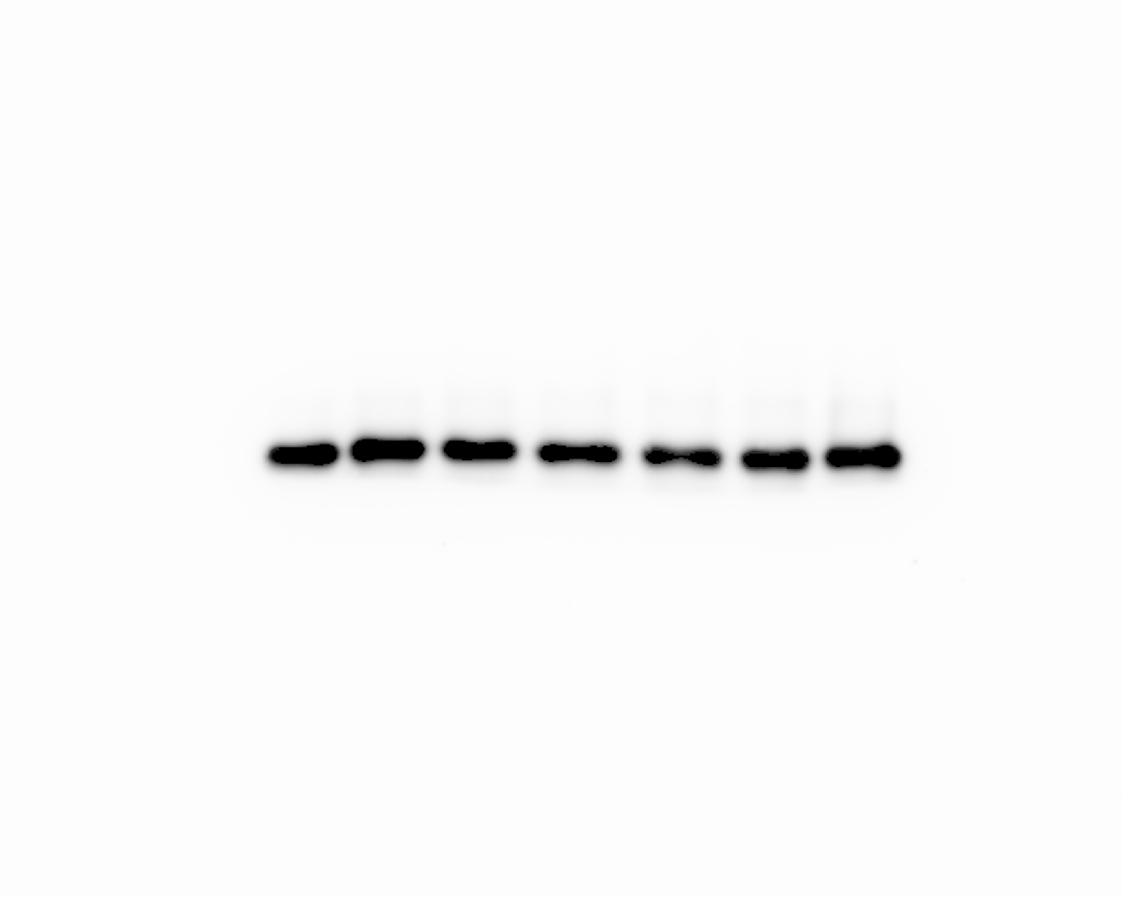

Supplement: Figure 6—source data 3. [file elife-103073-fig6-data3.zip › WB RAW NP AF/NP P21 #2(Chemiluminescence).tif]

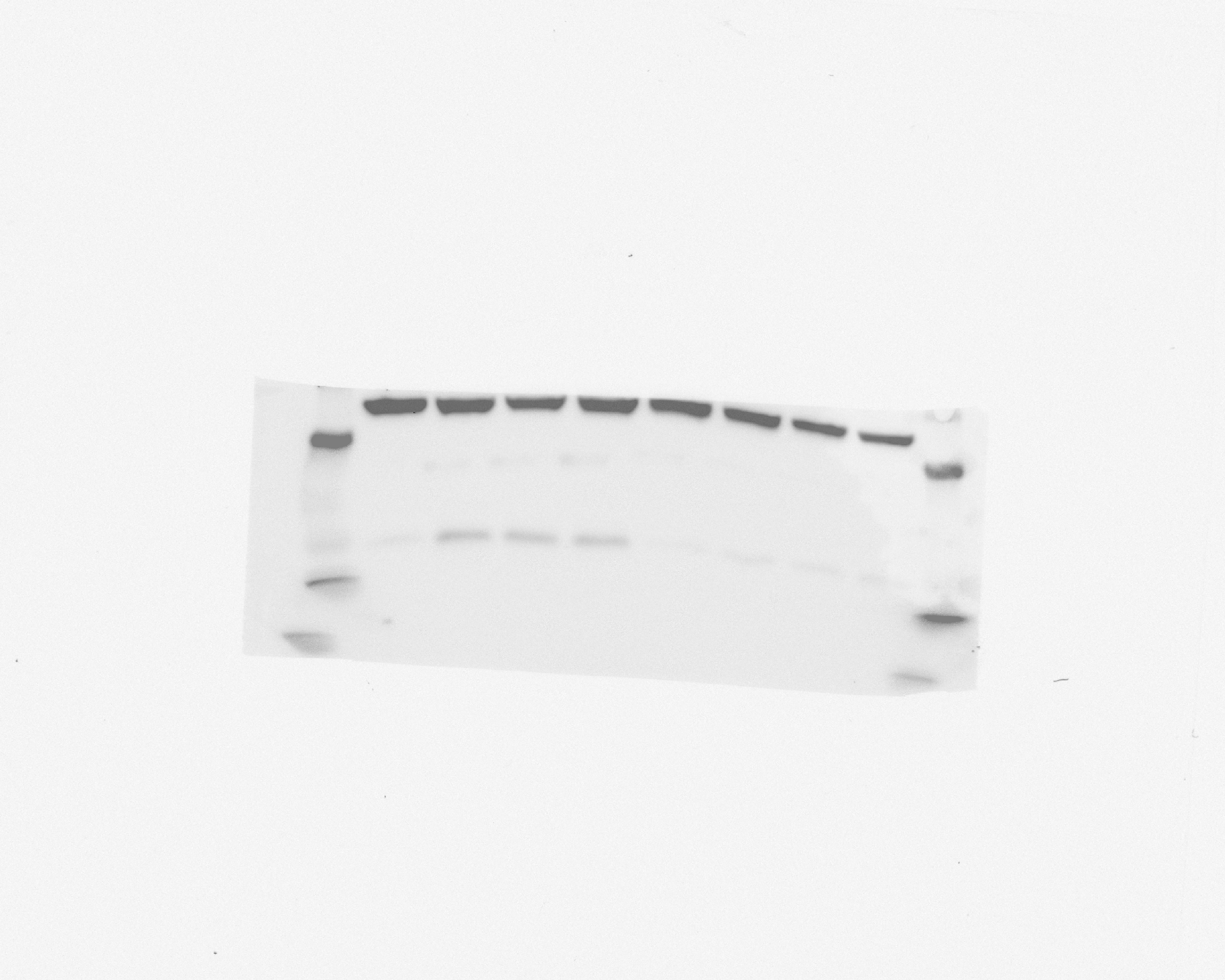

Supplement: Figure 6—source data 3. [file elife-103073-fig6-data3.zip › WB RAW NP AF/NP beta-actin #3(Composite).tif]

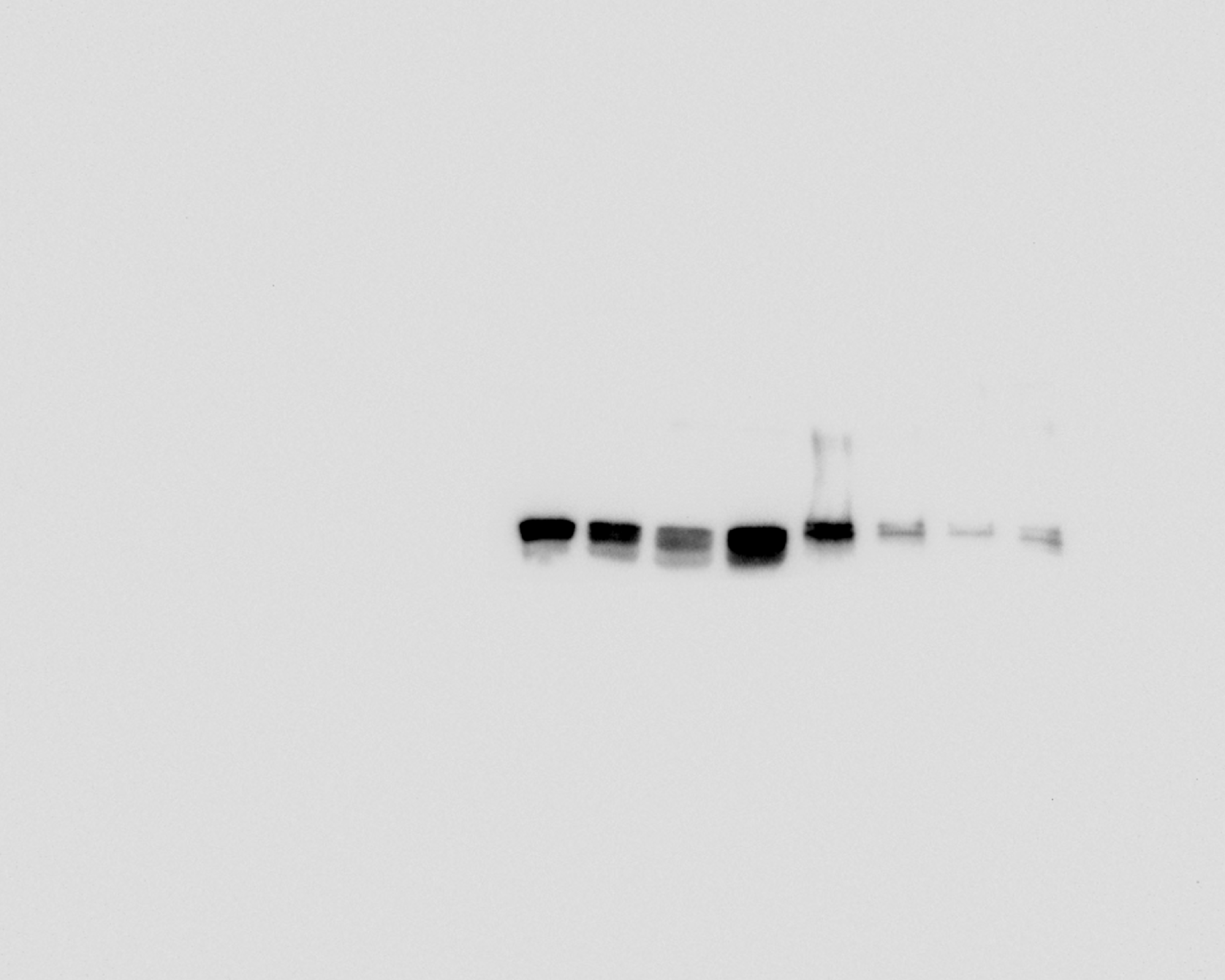

Supplement: Figure 6—source data 3. [file elife-103073-fig6-data3.zip › WB RAW NP AF/NP NFkb #4(Chemiluminescence).tif]

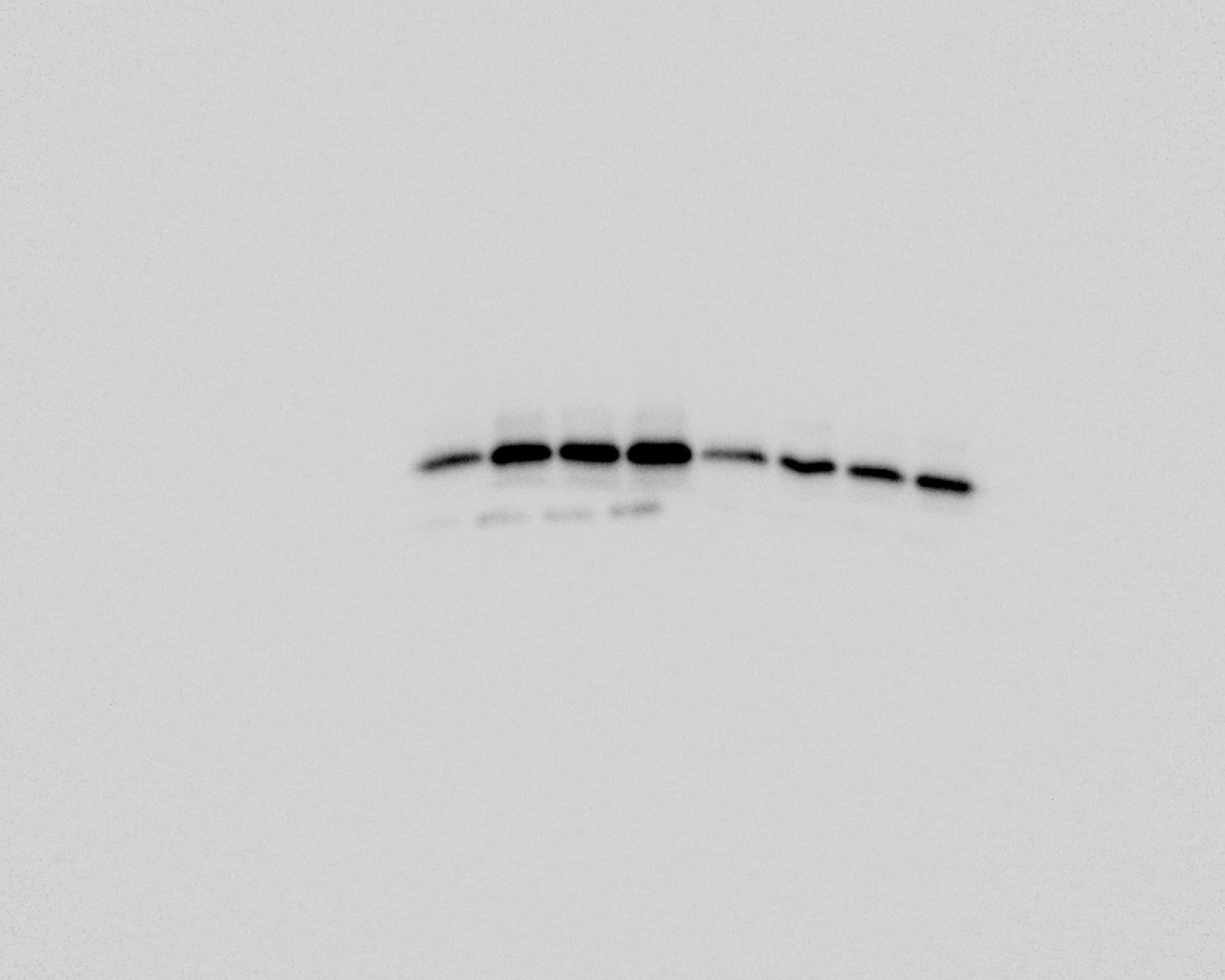

Supplement: Figure 6—source data 3. [file elife-103073-fig6-data3.zip › WB RAW NP AF/NP P21 #3(Chemiluminescence).tif]

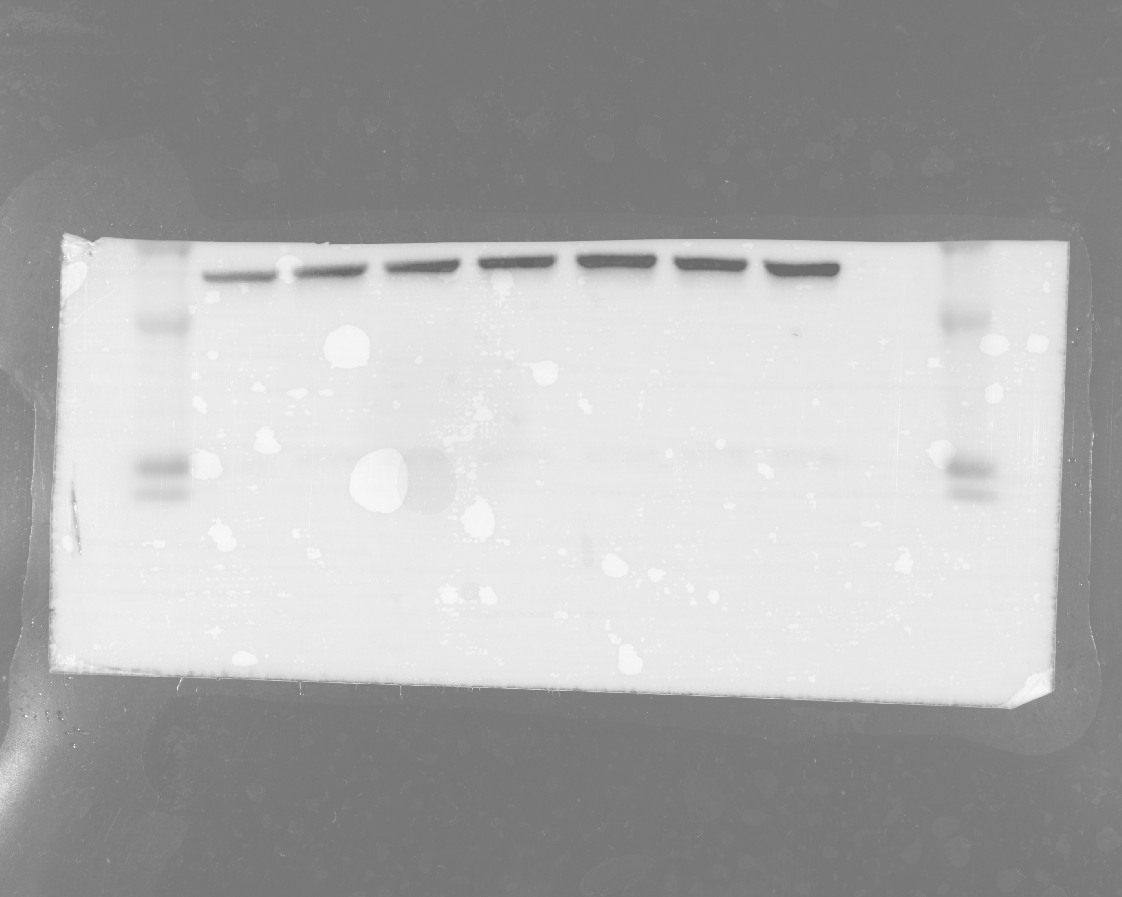

Supplement: Figure 6—source data 3. [file elife-103073-fig6-data3.zip › WB RAW NP AF/NP beta-actin #1(Composite).tif]

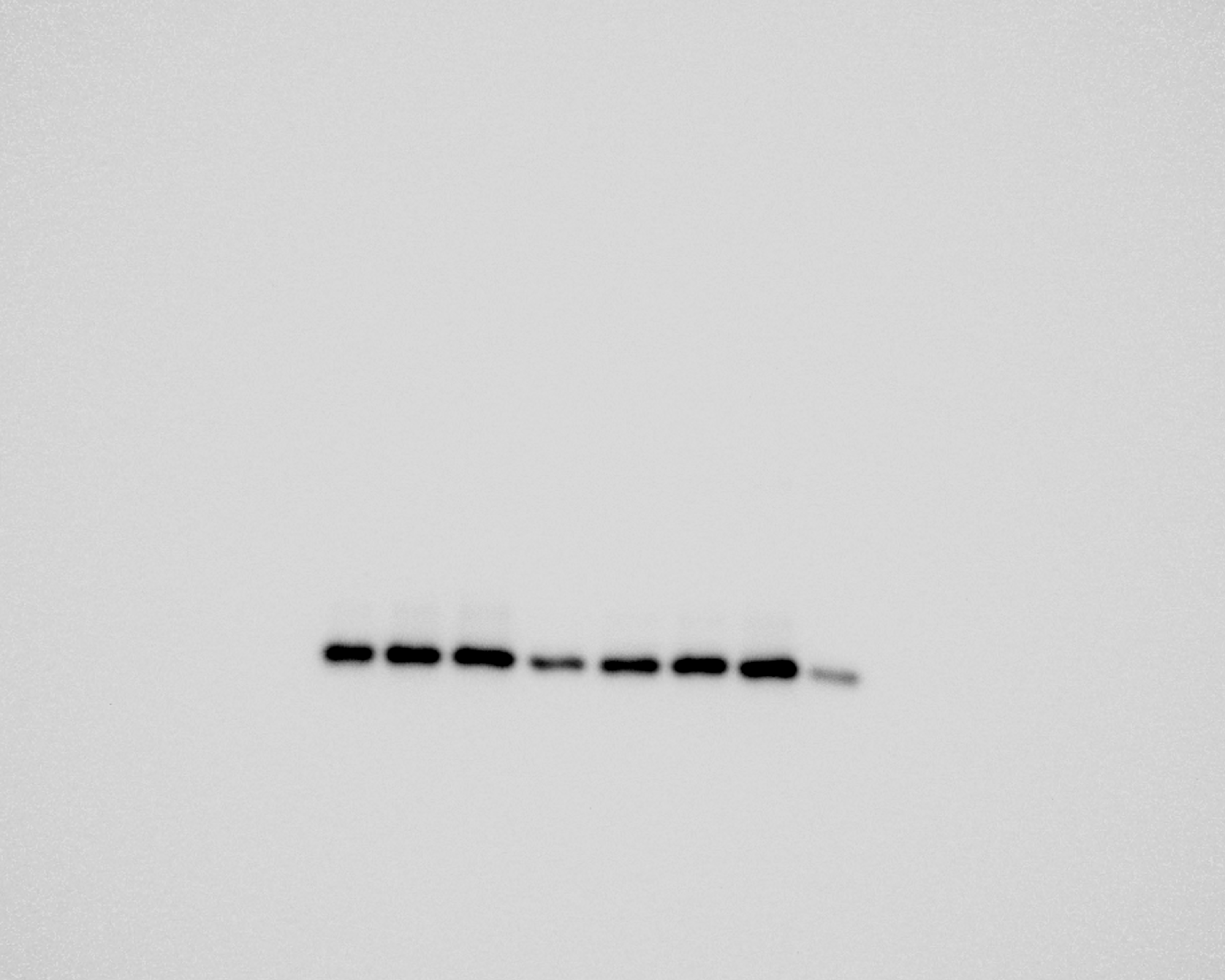

Supplement: Figure 6—source data 3. [file elife-103073-fig6-data3.zip › WB RAW NP AF/AF p21 #2(Chemiluminescence).tif]

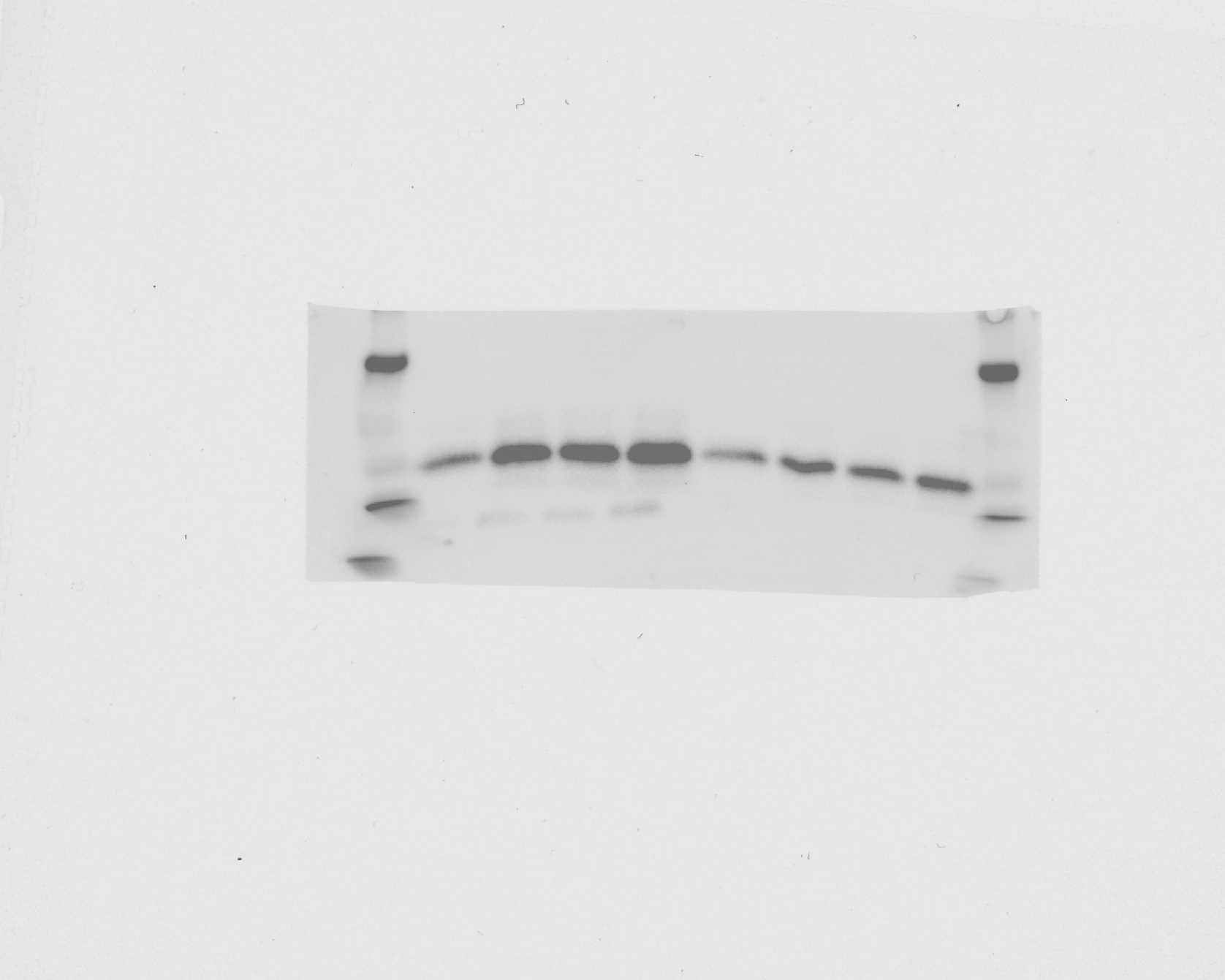

Supplement: Figure 6—source data 3. [file elife-103073-fig6-data3.zip › WB RAW NP AF/NP P21 #3(Composite).tif]

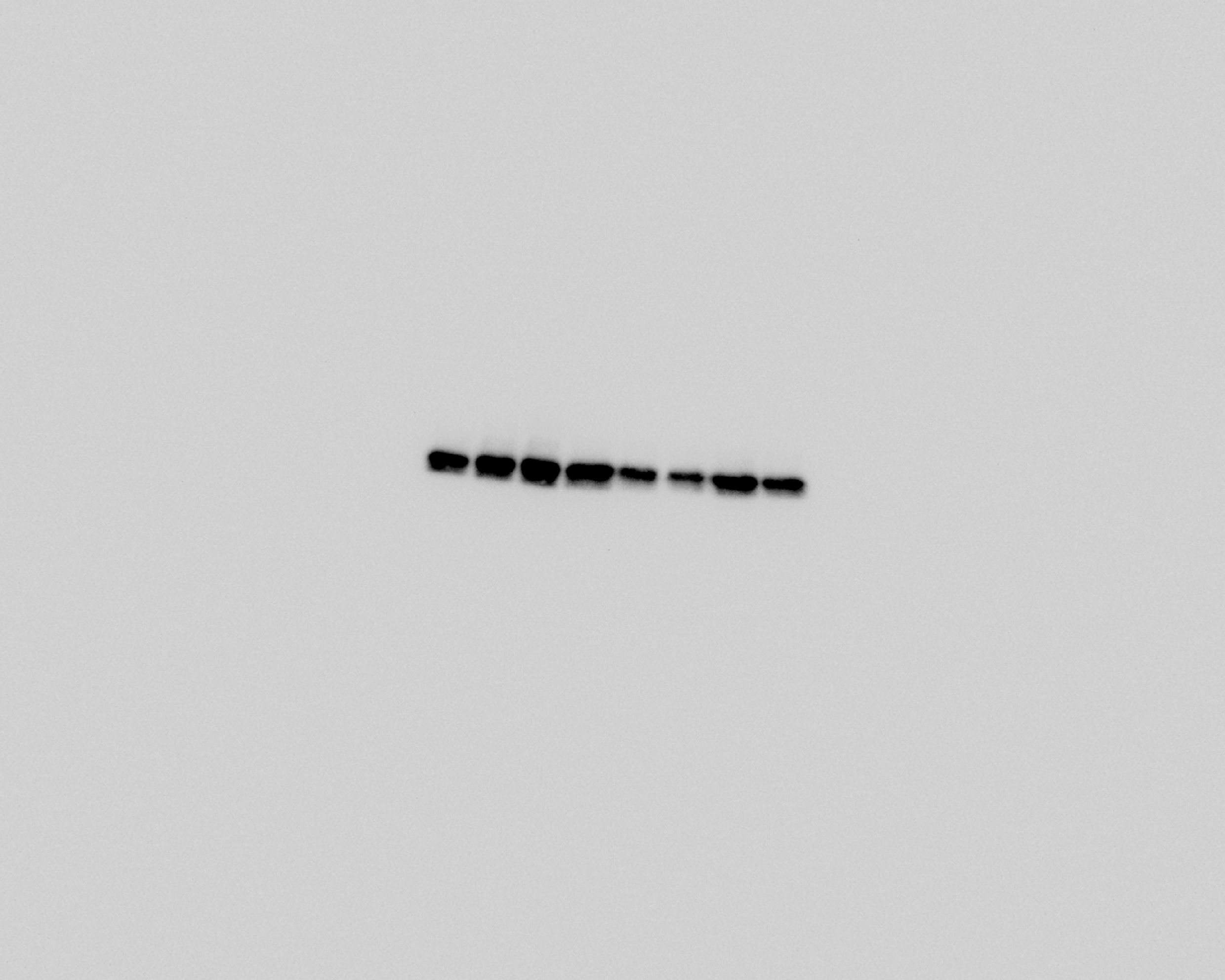

Supplement: Figure 6—source data 3. [file elife-103073-fig6-data3.zip › WB RAW NP AF/AF p21 #1(Chemiluminescence).tif]

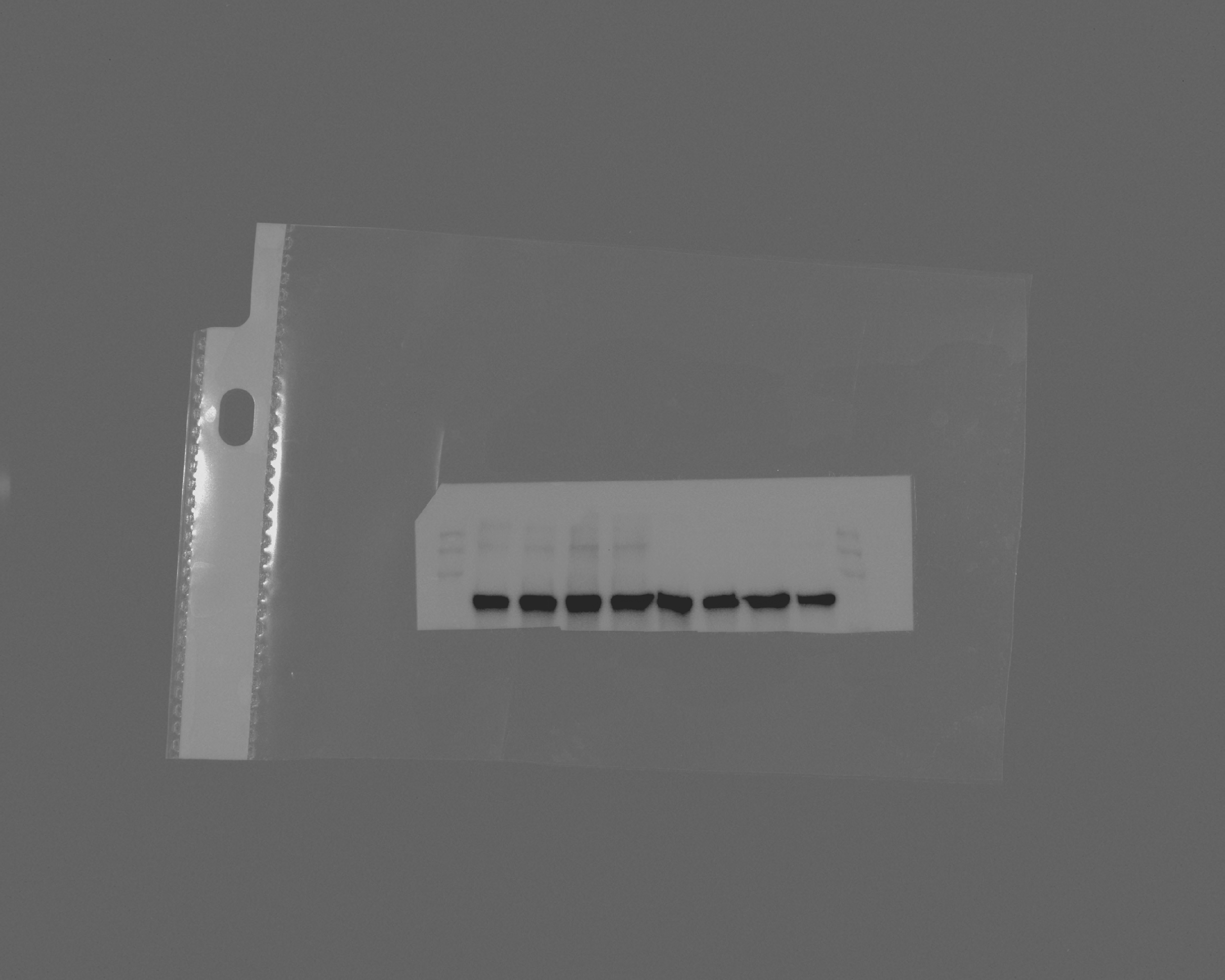

Supplement: Figure 6—source data 3. [file elife-103073-fig6-data3.zip › WB RAW NP AF/AF nfkb #1(Composite).tif]

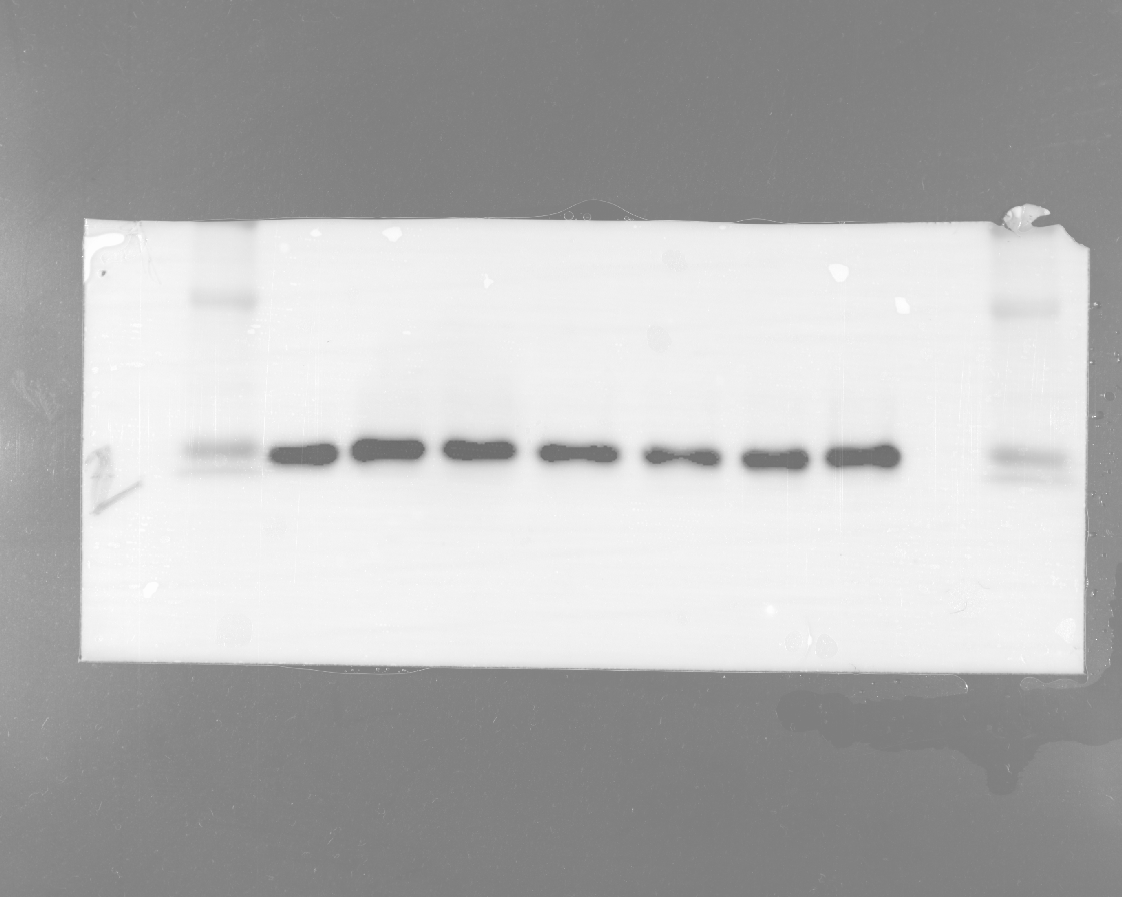

Supplement: Figure 6—source data 3. [file elife-103073-fig6-data3.zip › WB RAW NP AF/NP P21 #2(Composite).tif]
